# A Bayesian life-course linear structural equations model (BLSEM) to explore the development of body mass index (BMI) from the prenatal stage until middle age

**DOI:** 10.1038/s41366-025-01857-8

**Published:** 2025-08-20

**Authors:** Evangelia Tzala, Marco Banterle, Ville Karhunen, Tom A. Bond, Mimmi Tolvanen, Marika Kaakinen, Sylvain Sebert, Alex Lewin, Marjo-Riitta Jarvelin

**Affiliations:** 1 Department of Epidemiology and Biostatistics, MRC Centre for Environment Health, School of Public Health, Imperial College, London, UK; 2 Department of Medical Statistics, London School of Hygiene and Tropical Medicine, London, UK; 3 Research Unit of Population Health, Faculty of Medicine, University of Oulu, Oulu, Finland; 4 MRC Integrative Epidemiology Unit at the University of Bristol, Bristol, UK; 5 Population Health Sciences, Bristol Medical School, University of Bristol, Bristol, UK; 6 Frazer Institute, The University of Queensland, Brisbane, QLD Australia; 7 Faculty of Health and Medical Sciences, School of Biosciences, University of Surrey, Guildford, UK; 8 Institute for Molecular Medicine Finland, Helsinki Institute of Life Science, University of Helsinki, Helsinki, Finland; 9 Unit of Primary Health Care, Oulu University Hospital, OYS, Oulu, Finland; 10 Department of Life Sciences, College of Health and Life Sciences, Brunel University London, London, UK

**Keywords:** Epidemiology, Obesity

## Abstract

**Objective and methods:**

We have developed a novel Bayesian Linear Structural Equations Model (BLSEM) with variable selection priors (available as an R package) to build directed acyclic graphs to delineate complex variable associations and pathways to BMI development. Conditional on standard assumptions used in causal inference, the model provides interpretable estimates with uncertainty for natural direct, indirect (mediated) and total effects.

**Results:**

We showcase our method using data on 4119 offspring followed from the pre-pregnancy period to age 46 years (y) in a Finnish population-based birth cohort. The BLSEM enabled efficiently to analyse all available data over the long-time span, identifying factors to distil potential causal pathways contributing to adult BMI development. All of the associations between early childhood and adolescence variables with adult BMI at 46 y (BMI46) were indirect via multiple paths. For example, maternal prepregnancy BMI, smoking and socioeconomic position are associated with BMI46 through 35, 31 and 26 paths. Another notable feature was that the contribution of very early life factors, particularly prenatal, was captured by growth patterns along childhood, which were the strongest early predictors of middle age BMI46 (the age at adiposity rebound (AgeAR), early growth parameters between the AgeAR to 11 y). BMI and blood pressure measured 15 y earlier also predicted BMI46, all other factors held constant. Genetic predisposition by the polygenic risk score for BMI showed an indirect effect that became apparent at AgeAR and thereafter.

**Conclusions:**

The Bayesian approach we present and the BLSEM software developed advances methodologies for the analysis of complex, multifaceted life-course data prior to the estimation of potential causal pathways. Our results, although exploratory in nature, suggest that the effective interventions to tackle adverse BMI development could be designed throughout childhood, though the period by AgeAR may be paramount. We feature the importance of integrated life-course analyses that help to understand the contribution of life-stage factors of development.

## Introduction

Structural equation modelling (SEM) and path analysis are powerful multivariate statistical techniques that provide a flexible framework for analysing complex relationships among multiple variables, which may influence one another reciprocally and are intrinsically ordered over time, directly or indirectly through mediator variables [1]. They can be particularly useful in analyses of longitudinal information, where researchers need to identify and interpret the relationships in complex systems that may underlie disease development, ultimately aiming to make inferences of a causal nature of relationships [2, 3]. Methodologically, we have an urgent need to move from traditional statistical analysis in epidemiology to causal analysis of multifaceted data with emphasis on the assumptions that underlie causal inferences, and the conditional nature of causal and counterfactual claims [4]. Here, we showcase an exploratory Bayesian approach to longitudinal data. We build directed acyclic graphs (DAGs) for the variable relationships and start with a much larger number of potential risk or protective factors in the model than is usual in path analysis models. The method searches through all sets of variables’ associations, selecting the ones supported by the data, i.e. not specified a priori. The Bayesian (probabilistic modelling) approach includes uncertainty on the estimated DAG, in the form of inclusion probabilities for each arrow in the DAG, and provides interpretable estimates with uncertainty for direct and indirect (mediated) effects.

To date, to the best of our knowledge, this is the first time that a comprehensive model, Bayesian path analysis with variable selection, is developed and tested for these purposes and applied to explore simultaneously the interrelationships of a wide range of potentially correlated genetic and environmental risk factors for the development of BMI by middle age from the prenatal period.

We use BMI as a measure for obesity risk, one of the greatest long-term public health challenges of the twenty-first century [5, 6]. Bray et al. and the World Obesity Federation support defining person’s obesity as a chronic relapsing disease [7, 8]. Although this concept has sparked controversy in the last century, accepting it can focus attention on successfully tackling obesity and reducing the risk of declining life-expectancy and many of its associated chronic disease co-morbidities, such as type 2 diabetes, cardiovascular disease and certain cancers [9, 10]. Moreover, the association between obesity and infectious diseases has received increasing recognition over the last years e.g. due to the 2009 pandemic influenza A (H1N1) and Coronavirus Disease (COVID-19) [11, 12].

An extensive number of multidimensional risk factors, potentially age-dependent, e.g. genetic, molecular, social, environmental, are associated with obesity development. Understanding their relationships but also how individuals may develop, grow and change throughout their lives, from the prenatal period onwards, is key to inform possible interventions. Observational and genetic studies show that there are important postnatal age-related stages for the development of diseases, such as periods around adiposity peak (AP) in infancy, adiposity rebound (AR) at pre-school age and puberty. During these stages, individuals may be more susceptible to the impact of external factors, and the genetic influences of BMI may vary [13–15].

We utilised the extensive follow-up of the Northern Finland Pregnancy Birth Cohort 1966 (NFBC1966) [16] and its detailed data on childhood growth, motivated by the consistent findings reported in the literature that growth patterns at infancy, childhood and puberty are related to later adiposity [14, 15, 17, 18], to model growth patterns across the life-course together with other essential data. We undertook the Bayesian path analysis model (Bayesian Linear SEM, BLSEM) with variable selection aiming to (i) develop and test life-course model methods to explore the network of factors associated with BMI development considering the time ordering, (ii) examine how growth over childhood and adolescence, in particular specified growth parameters (age and BMI at the AP and AR) link with later BMI and (iii) understand critical periods potentially for early intervention.

## Materials and methods

### Data description

The study population is part of the prospective, longitudinal, population-based Northern Finland Pregnancy Birth Cohort 1966 (NFBC1966), which represents a relatively genetically and environmentally homogeneous sample with a high coverage of 96% of all births in the two northernmost provinces of Finland in 1966. The NFBC1966 has been described elsewhere [16].

Since birth, individuals have been followed up with postal questionnaires with questions on demographic, health, lifestyle and socio-economic indicators and/or clinical examinations with blood samples and anthropometric measurements at 1, 14, 31, and 46 years (y). Information on the mothers was retrieved from structured self-administered questionnaires completed at maternity clinics. Women entered the antenatal communal care usually on average by the 16th gestational week. Pre-pregnancy and course of pregnancy data were collected by midwives in the clinics on the standard forms. These data were further transferred into study databases. Birth data were collected from the hospital records after each delivery. These data have been supplemented with repeated childhood growth measurements (an average of 20 weight and height measurements from early infancy to late adolescence) collected by nurses at welfare clinics as part of the national child-health screening programme that is free and available for all children born in Finland (overall 100% attendance). Childhood growth data have been used to derive growth parameters at AP around 9 months and childhood AR point around 5.5 y [19].

For the present study, we included individuals with complete data on growth parameter measurements at AP and AR. Though we imputed other missing variables, we found there was not enough information in the data to be able to impute missing values of the growth parameters within our model. Excluding multiple births, a total of 4119 offspring, 2154 (52.3%) males and 1965 (47.7%) females were included in the analysis. Supplementary Fig. S1 shows the study flowchart and Fig. S2 study’s geographical location.

**Fig. 1:**
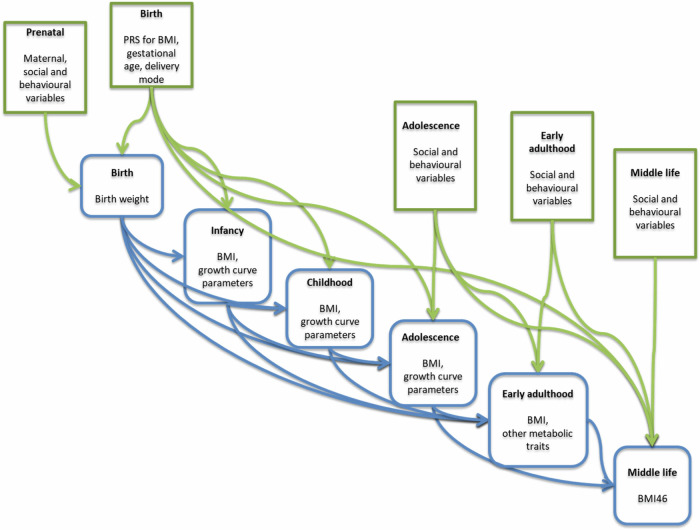
Simplified conceptual directed acyclic graph (DAG) showing the model and the relationships between the variables. The arrows in the model represent potential directions of the associations. Green boxes represent exogenous variables (i.e. predictors only) and blue boxes represent endogenous variables (i.e. both life-stage-specific outcomes and predictors). Genetic and lifestyle variables are considered statistically as exogenous, whilst anthropometric, growth and metabolic health variables are treated as endogenous. Endogenous and exogenous variables are introduced in Table 1, whilst a detailed description of all variables in this analysis is provided in Supplementary Table S1.

For the purposes of the path analysis model, variables (endogenous and exogenous) are grouped into blocks, corresponding to life stages. Endogenous variables appear in the model as intermediate outcomes at one stage and explanatory variables at the next stage (blue boxes in Fig. 1) whilst exogenous variables are explanatory variables in all stages (green boxes in Fig. 1). Anthropometric and metabolic traits and growth parameters are considered endogenous variables due to the well-established evidence that they have been linked to obesity and other health indicators in adult life [19–21]. The remaining genetic and lifestyle variables are treated as exogenous exposures in this analysis. Table 1 introduces endogenous and exogenous variables by life stage, and Fig. S3 shows the correlation matrix between the variables in the analyses.

**Table 1:** Glossary table for BLSEM of endogenous variables that can be both life-stage-specific outcomes and predictors (in bold), as well as of exogenous variables that are predictors only.

Life-stage (block in Fig. 2)	Endogenous variables	Endogenous (in bold) and exogenous variables
Prenatal (pre-pregnancy and pregnancy), birth	Birth weight (BW)	Maternal pre-pregnancy BMI (matBMI), maternal age (matAGE), socio-economic status of the family or mother (matSEP), number of children the mother has including this child (parity), maternal marital status at birth (matMAR), maternal residence in city, small town or rural centre (town), or in remote village(village), maternal smoking at the 2nd month of pregnancy (matSMO), number of people in the household (houseN), maternal wealth indicator (matWEALTH), gestational hypertension (matGH), chronic hypertension (matCHP), pre-eclampsia & super-imposed pre-eclampsia (matPE), systolic/diastolic blood pressure elevated (matSBP, matDBP), hypertension not determined or not known (matHPNA), paternal age (patAGE), polygenic risk score for BMI (PRSBMI), gestational age (gestAGE), caesarean section (caesarean), placental weight (placWHT).
At infancy (1 y)	Age at Adiposity Peak (AgeAP), BMI at AP (BMIAP), Mean BMI velocity birth-AP (BMIvelAP), Peak height velocity (PHV)	matBMI, matAGE, matSEP, parity, matMAR, town, village, matSMO, houseN, matWEALTH, matGH, matCHP, matPE, matSBP, matDBP, matHPNA, patAGE, PRSBMI, gestAGE, caesarean, placWHT, **BW**.
At childhood (6 y)	Age at Adiposity Rebound (AgeAR), BMI at AR (BMIAR), Mean BMI velocity AP-AR (BMIAPAR), Mean BMI velocity AR-11y (BMIAR11)	matBMI, matAGE, matSEP, parity, matMAR, town, village, matSMO, houseN, matWEALTH, matGH, matCHP, matPE, matSBP, matDBP, matHPNA, patAGE, PRSBMI, gestAGE, caesarean, placWHT, **BW,** **AgeAP,** **BMIAP,** **BMIvelAP,** **PHV**.
At 14 y	BMI at 14 y (BMI14), Mean BMI velocity 11y-15y (BMI11-15)	matBMI, matAGE, matSEP, parity, matMAR, town, village, matSMO, houseN, matWEALTH, matGH, matCHP, matPE, matSBP matDBP, matHPNA, patAGE, PRSBMI, gestAGE, caesarean, placWHT, **BW,** **AgeAP,** **BMIAP,** **BMIvelAP,** **PHV, AgeAR, BMIAR, BMIAPAR, BMI AR11**.
At 31 y	BMI (BMI31), Blood pressure latent factor (BPF31), Insulin (INS31), Waist circumference (WC31), High-density lipoprotein cholesterol (HDL31), Low-density lipoprotein cholesterol (LDL31), Triglycerides (TRIGL31)	matBMI, matAGE, matSEP, parity, matMAR, town, village, matSMO, houseN, matWEALTH, matGH, matCHP, matPE, matSBP, matDBP, matHPNA, patAGE, PRSBMI, gestAGE, caesarean, placWHT, **BW, AgeAP, BMIAP, BMIvelAP, PHV, AgeAR, BMIAR, BMIAPAR, BMIAR11, BMI14, BMI11-15**, smoking (SMO14), alcohol use (ALCO14), physical activity (PA14), socio-economic status (SEP14), smoking (SMO31), smoking pack years (PACKYR31), alcohol use (ALCO31), diet (DIET31), physical activity (PA31), number of adults in the household (ADULT31N), number of children in the household (CHILD31N), socio-economic status latent factor (SEPF31), socio-economic status (SEP31), psychosocial latent factor (PSCF31).
At 46 y	BMI (BMI46)	matBMI, matAGE, matSEP, parity, matMAR, town, village, matSMO, houseN, matWEALTH, matGH, matCHP, matPE, matSBP, matDBP, matHPNA, patAGE, PRSBMI, gestAGE, caesarean, placWHT, **BW, AgeAP, BMIAP, BMIvelAP, PHV, AgeAR, BMIAR, BMIAPAR, BMIAR11, BMI14, BMI11-15**, smoking (SMO14), alcohol use (ALCO14), physical activity (PA14), socio-economic status (SEP14), smoking (SMO31), smoking pack years (PACKYR31), alcohol use (ALCO31), diet (DIET31), physical activity (PA31), number of adults in the household (ADULT31N), Number of children in the household (CHILD31N), socio-economic status latent factor 1 (SEPF31), socio-economic status latent factor 2 (SEP31), psychosocial latent factor (PSCF31), **BMI31, BPF31, INS31, WC31, HDL31, LDL31, TRIGL31**, smoking (SMO46), alcohol use (ALCO46), diet (DIET46), socio-economic status latent factor at 46 y (SEP46).

Abbreviations, mainly for figures, are shown in brackets per life stage (*n* = 4119). A detailed description of each variable is provided in Supplementary Table S1.

### Endogenous variables (i.e. both life-stage-specific outcomes and predictors)

Growth in utero was represented by birth weight, as this is a more accurate measure than BMI at this stage, while considering gestational age. For postnatal growth indices, we used BMI at AP and AR from fitted growth curves and focussed on BMI between these phases and other important periods of growth, including prepubertal (before 11 y) and pubertal (11–15 y). The selection of 11 and 15 y cut points was based on the best availability of data as well as biological growth. We calculated mean growth velocities for BMI between (1) birth and AP, (2) AP and AR, (3) AR and 11 y, (4) 11 and 15 y. BMI at AP and AR were selected (rather than BMI at fixed ages in infancy and childhood) because previous evidence suggests that these measures play an important role for relevant health outcomes in adulthood [19, 21–24]. For adolescence, we used BMI at 14 y of age (before age 15 y). The methods used for growth modelling of BMI and age have been described in detail by Sovio et al. [19].

For early adulthood at 31 y, we used anthropometric (BMI, waist circumference) and metabolic health (insulin, triglycerides, HDL- and LDL-cholesterol) measurements. Moreover, we included a latent factor to represent blood pressure based on diastolic and systolic blood pressure measurements [25]. BMI at 46 y, an indicator of body mass and obesity in later life, was taken as the distal outcome. Trained research nurses performed the anthropometric measurements at the clinical examinations at ages 31 and 46 y.

### Exogenous variables (i.e. predictors only)

A Polygenic Risk Score (PRS) for adult BMI was used as an explanatory variable in the model from birth onwards. The BMI PRS was calculated as a weighted sum of BMI-increasing alleles at 591,827 single-nucleotide polymorphisms (SNPs) across the genome. For the calculation of SNP weights, we used the BOLT-LMM linear predictor [26] and estimated BOLT-LMM SNP effects in the UK Biobank data [27, 28]. Full details of the calculation of PRS can be found in previously published work [29].

Variables at prenatal period or at birth consisted of maternal pre-pregnancy BMI, maternal age, smoking at the second month of pregnancy, hypertensive disorders during pregnancy, marital status, residence, a latent factor for familial socio-economic position, wealth index, paternal age, gestational age at birth, mode of delivery and placental weight. A detailed description of all the variables in the analysis is provided in Supplementary Table S1.

For adolescence at 14 years of age, we used self-reported information on adolescent’s smoking habits (non-smoker/occasional-regular smoker) [30], alcohol consumption (non-consumer/regular consumer) [31], physical activity (less than once a week/once a week or more doing sports after school hours) [32] and a latent factor for familial socio-economic status (Table S1). For early adulthood at 31 y, we included smoking habits (non-smoker/occasional-regular smoker) [30], smoking pack years (number of packs smoked in a day divided by 20 × years of smoking for current smokers), alcohol consumption (grams/day) [33], diet score [34], physical activity as metabolic equivalent (MET) hours/week [32] number of adults and children in the household, a psychosocial latent factor reflecting psychosocial wellbeing of the participant [25] and two latent factors for own socio-economic status [25]. The latent factors were developed to combine groups of highly correlated variables into composite variables. Using these composite variables in place of the separate correlated variables results in more stable and reliable estimates (in common with all regression-based analyses).

For middle-age adulthood at 46 years, we used smoking habits (non-smoker/occasional-regular smoker) [30], alcohol consumption (grams/day), diet score [34] and a latent factor for own socio-economic status (Table S1).

### Bayesian linear structural equations model (BLSEM)

We used a path analysis approach to model the longitudinal development of BMI and other growth parameters over six life stages (from birth up to middle life). We started by constructing a DAG connecting the six life stages in chronological order (Fig. 1), with a set of endogenous variables and a set of exogenous variables at each life stage (see Table 1 for full lists of all endogenous and exogenous variables).

We used a Bayesian linear structural equations model (BLSEM) to model the relations amongst all variables. An arrow present in the DAG in Fig. 1 pointing forward from a block to the next one means that in the BLSEM, we allowed every variable in the former block to potentially appear as a covariate in a regression model for every variable in the latter block. Endogenous variables at earlier life stages were allowed to appear as covariates in regressions for later life stages.

We used variable selection priors to find subsets of the covariates in each regression model. Hence, whilst we started with a large number of variables in the analysis, we ended up estimating a sparser DAG. Full details of the model and estimation of potential causal pathways in the model are given in the Supplementary material.

### Imputation model

In the Bayesian modelling framework, missing values are treated as unknown quantities; that is, they appear as parameters in the model, which are predicted as part of the model estimation process. In our analysis, all variables are assumed to be missing at random (MAR), i.e. missing values can be predicted from observed data. Missing values in endogenous variables are predicted from the posterior predictive distributions of the regression models [35]. For missing exogenous variables, the imputation model consists of a joint multivariate distribution over all exogenous covariates. In our analysis, the joint model for exogenous variables is the multivariate Normal/Probit, with categorical variables being modelled as thresholded versions of latent Normal variables.

### Model output

Using Bayesian estimation, we obtained the joint posterior distribution for all parameters in the model, including both regression coefficients and inclusion probabilities for each covariate in each regression in the BLSEM. To summarise the posterior on the regression coefficients, we used mean or median point estimates and posterior credible intervals. In order to see how much of the variation in each endogenous variable is explained by dependence on the exogenous variables, we obtain a Bayesian version of *R*-squared for each endogenous variable. Details and formula for the Bayesian *R*-squared are given in the Supplementary material.

**Fig. 2:**
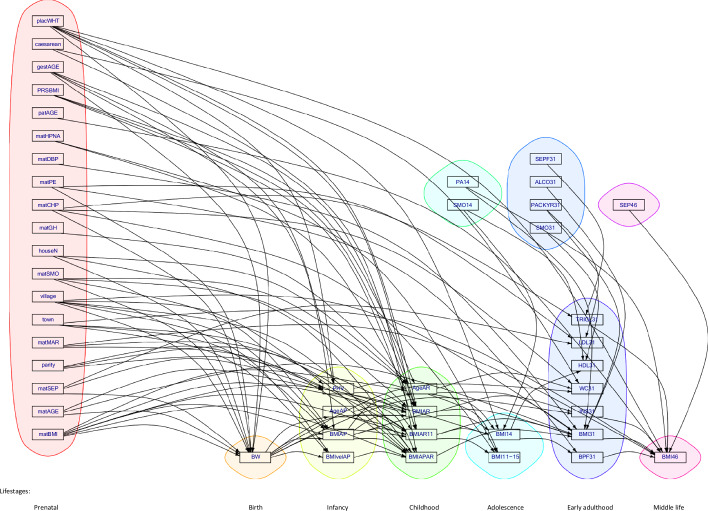
Directed acyclic graph (DAG) from the Bayesian path analysis model (BLSEM) on BMI46 thresholding for mean posterior probabilities (MPPIs) ≥ 0.5 to illustrate multiple pathways across the life-course (*n* = 4119). A glossary for the variable names is provided in Table 1 and further description of the variables in Supplementary Table S1. Table 3 shows standardised effect sizes (*β*s) × 10^3^.

For the variable selection, we used the marginal posterior probabilities of inclusion (MPPI) for variable j in response k of block q. These are model-averaged probabilities of association between variables *j* and *k* or summaries of uncertainty about the strength of the association between the two variables. To visualise the results, we constructed a simplified graph as follows: if the MPPI is 0.5 or greater, we include an arrow from variable *j* to variable *k*. The resulting graph is shown in Fig. 2.

## Results

### Descriptives of the data

Table 2 summarises the distributions of the anthropometric, growth and metabolic health variables by sex. Supplementary Tables S1 and S2 describe in detail all the other variables in the model.

**Table 2:** Characteristics of the anthropometric, growth and metabolic health variables, the so-called ‘endogenous’ variables, used in the present analysis.

	Males (*N* = 2154)	Females (*N* = 1965)	Total (*N* = 4119)
Characteristic	Mean (SD)	Mean (SD)	Mean (SD)
BW at birth, kg	3.55 (0.53)	3.43 (0.50)	3.49 (0.52)
Age at AP, y	0.76 (0.03)	0.76 (0.03)	0.76 (0.03)
BMI at AP, kg/m^2^	18.20 (0.79)	17.80 (0.78)	18.01 (0.82)
Mean BMI velocity birth-AP, (kg/m^2^)/y	5.92 (1.73)	5.41 (1.70)	5.67 (1.73)
Peak height velocity, cm/y	54.35 (3.20)	50.82 (3.90)	52.60 (3.94)
Age at AR, y	5.68 (0.89)	5.54 (0.93)	5.62 (0.91)
BMI at AR, kg/m^2^	15.50 (1.03)	15.40 (1.14)	15.44 (1.08)
Mean BMI velocity AP-AR, (kg/m^2^)/y	−0.54 (0.17)	−0.50 (0.19)	−0.52 (0.18)
Mean BMI velocity AR-11y, (kg/m^2^)/y	0.33 (0.21)	0.35 (0.22)	0.34 (0.21)
BMI at 14 y, kg/m^2^	19.23 (2.63)	19.50 (2.58)	19.36 (2.61)
Mean BMI velocity 11–15 y, (kg/m^2^)/y	0.58 (0.41)	0.71 (0.45)	0.64 (0.43)
BMI at 31 y, kg/m^2^	25.25 (3.39)	23.64 (4.25)	24.45 (3.93)
Mean systolic blood pressure at 31 y, mmHg, SBP^a^	130 (13)	120 (12)	125 (13)
Mean diastolic blood pressure at 31 y, mmHg, DBP^a^	80 (11)	75 (11)	78 (11)
Insulin at 31 y, μIU/mL	9.04 (5.12)	8.68 (7.21)	8.78 (4.95)
Waist circumference at 31 y, cm	89.07 (9.76)	79.11 (12.27)	84.14 (12.14)
HDL-C at 31 y, mmol/L	1.41 (0.33)	1.69 (0.38)	1.55 (0.38)
LDL-C at 31 y, mmol/L	3.24 (0.91)	2.81 (0.82)	3.02 (0.89)
Triglycerides at 31 y, mmol/L	1.34 (0.81)	1.07 (0.60)	1.21 (0.72)
BMI at 46 y, kg/m^2^	27.40 (4.39)	26.62 (5.17)	26.98 (4.84)

Endogenous variables are dependent variables that can also be independent in later life stages of the model.

*BW* birth weight, *BMI* body mass index, *AP* adiposity peak, *AR* adiposity rebound, *HDL* high-density lipoprotein cholesterol, *LDL* low-density lipoprotein cholesterol.

^a^Analysed as a combined latent factor score (BPF31, Table S1).

### Direct, indirect and total effects from the Bayesian LSEM

We report results from the analysis conducted on the whole study sample. Analyses conducted separately for males and females showed no indication of moderating effects by sex (data not shown). All regressions in the model were adjusted for sex since this is generally seen to be associated with the life-course development of obesity [36].

The estimated path analysis diagram (DAG) for MPPI ≥ 0.5 is illustrated for multiple paths in Fig. 2. More conservative thresholds e.g. 0.8 (80%) or 0.9 (90%), may be used. We assumed no interaction between exposure and mediator variables and estimated direct, indirect and total effects. Table 3 shows the standardised (in SD units) direct, total indirect and total effects, together with the 95% credible intervals (CIs), and the number of paths for every variable in the model linked to BMI46. The standardised direct estimates (and 95% CI) on all endogenous variables in the BLSEM are provided in supplementary Table S3. Standardised effects are used to compare the relative importance of the endogenous and exogenous variables since they describe the change in the outcomes in SD units per a 1-SD change in the continuous predictors and per the change from 0 to 1 in the binary predictors, accounting for all factors in the model. Because of some small standard effects, estimates are presented in 10^3^ scale (Table 3).

**Table 3:** Results from the Bayesian path analysis model, BLSEM, for BMI46 (*n* = 4119).

Variables (unit/category)	Direct effect (×10^3^) and 95% CI	Total indirect effect (×10^3^) and 95% CI	Total effect (×10^3^) and 95% CI	No of paths
**Prenatal**				
Maternal pre-pregnancy BMI (kg/m^2^)		52.3 (41.7, 63.0)	52.3 (41.7, 63.0)	35
Maternal age (years)		−6.66 (−25.3, 3.91)	−6.66 (−25.3, 3.91)	27
SEP of the family (latent factor)		−1.85 (−3.57, −0.18)	−1.85 (−3.57, −0.18)	26
Parity		−12.7 (−21.8, −2.49)	−12.7 (−21.8, −2.49)	11
Maternal marital status at birth				
Not married	Referent			
Married	−31.6 (−206.1, 118.1)	−1.79 (−24.3, 15.15)	−33.4 (−205.0, 121.4)	4
Maternal place of residence				
City	Referent			
Small town and rural centre	13.5 (−44.4, 77.1)	2.83 (−7.20, 11.2)	16.4 (−40.5, 81.8)	11
Remote village		19.4 (−4.53, 60.6)	19.4 (−4.53, 60.6)	37
Maternal smoking				
Non-smoker	Referent			
Continued/stopped		22.5 (5.34, 41.0)	22.5 (5.34, 41.0)	31
Number of people in the household		−19.3 (−29.7, −9.69)	−19.3 (−29.7, −9.69)	22
Maternal hypertension				
Normotensive	Referent			
Gestational hypertension		5.04 (−15.1, 25.0)	5.04 (−15.1, 25.0)	2
Chronic hypertension		−1.31 (−4.98, 1.56)	−1.31 (−4.98, 1.56)	20
Pre-eclampsia (PE) and Super-imposed PE		−1.42 (−14.1, 17.2)	−1.42 (−14.1, 17.2)	20
Diastolic BP elevated		7.44 (−34.1, 61.8)	7.44 (−34.1, 61.8)	1
Could not be determined/ Not known		0.08 (−0.22, 0.58)	0.08 (−0.22, 0.58)	2
Paternal age (years)	−19.0 (−58.1, 5.7)		−19.0 (−58.1, 5.7)	1
**At birth**				
BMI PRS		14.8 (2.66, 22.8)	14.8 (2.66, 22.8)	6
Gestational age		0.85 (−2.38, 5.37)	0.85 (−2.38, 5.37)	36
Operative managements in delivery				
Vaginal, other	Referent			
Caesarean section		1.17 (−0.15, 7.53)	1.17(−0.15, 7.53)	1
Placental weight	5.95 (−21.8, 37.8)	4.02 (−1.32, 8.71)	9.97 (−18.0, 42.3)	32
Birth weight		17.9 (5.5, 29.3)	17.9 (5.5, 29.3)	19
**At infancy (1** **y)**				
Mean BMI growth velocity between birth and AP		−1.11 (−3.75, 0.00)	−1.11 (−3.75, 0.00)	1
BMI at AP		53.1 (18.7, 89.4)	53.1 (18.7, 89.4)	8
Age at AP		8.92 (0.00, 15.4)	8.92 (0.00, 15.4)	5
Peak height velocity		24.0 (−0.08, 37.9)	24.0 (−0.08, 37.9)	7
**At childhood (6** **y)**				
Mean BMI growth velocity between AP and AR		28.4 (0.00, 63.1)	28.4 (0.00, 63.1)	1
Mean BMI growth velocity between AR and 11 y		95.6 (39.1, 158.0)	95.6 (39.1, 158.0)	2
BMI at AR		4.46 (0.00, 9.19)	4.46 (0.00, 9.19)	2
Age at AR		−174.0 (−219.0, −124.3)	−174.0 (−219.0, −124.3)	2
**At adolescence (14** **y)**				
Smoking at 14 y		0.00 (−0.50, 0.48)	0.00 (−0.50, 0.48)	1
Physical activity at 14 y	22.6 (−52.6, 97.0)	1.75 (−28.0, 36.2)	24.3 (−54.1, 101.7)	2
BMI at 14 y		7.81 (0.00, 14.5)	7.81 (0.00, 14.5)	1
**At early adulthood (31** **y)**				
Smoking pack years at 31 y		−2.41 (−18.9, 9.39)	−2.41 (−18.9, 9.39)	1
Alcohol use at 31 y		13.9 (−0.06, 35.4)	13.9 (−0.06, 35.4)	1
SEP latent factor-1 at 31 y		0.29 (−0.56, 2.26)	0.29 (−0.56, 2.26)	1
Blood pressure latent factor at 31 y	80.4 (46.1, 113.8)		80.4 (46.1, 113.8)	1
BMI at 31 y	539.3 (500.3, 577.9)		539.3 (500.3, 577.9)	1
Insulin at 31 y	25.5 (−8.20, 66.3)		25.5 (−8.20, 66.3)	1
Triglycerides at 31 y	10.4 (−15.6, 49.6)		10.4 (−15.6, 49.6)	1
**At middle life (46** **y)**				
SEP latent factor at 46 y	−19.8 (−48.4, 7.00)		−19.8 (−48.4, 7.00)	1

Effect sizes (*β*s) are reported in SD units of BMI (kg/m^2^) by exposure unit, SD or category. Analysis was adjusted for sex. Direct effects are reported for associations with MPPI ≥ 0.5. Indirect effects are reported for paths through the DAG where every link on the path has MPPI ≥ 0. 5. Findings for MPPI < 0.5 are not shown.

Growth velocities: kg/m^2^/year.

*MPPIs* mean posterior probabilities of inclusion, *CI* Credible Intervals, *BMI* body mass index, *SEP* socio-economic position, *PRS* polygenic risk score, *AP* adiposity peak, *AR* adiposity rebound, *WC* waist circumference, *HDL* high-density lipoprotein cholesterol, *LDL* low-density lipoprotein cholesterol.

43 out of the initial 59 variables were selected (MPPI ≥ 0.5), with over 700 associations discovered by the model that illustrate the demands for the study/data, complexity of modelling and associations. Of prenatal factors, maternal BMI had amongst the largest positive standardised effects, equal to 52.3 (41.7 to 63.0) × 10^−3^ SD units of BMI46 (corresponding to 0.08 (0.06 to 0.10) kg/m^2^), accounting for all possible mediation through growth in the life-course (35 paths, subgraph in Fig. S4). Similarly, maternal smoking was indirectly associated through 31 paths (Fig. S5) with 22.5 (5.34 to 41.0) × 10^−3^ SD units (corresponding to 0.03 (0.008 to 0.06) kg/m^2^) higher BMI46 in the offspring of smoker mothers compared with the offspring of non-smoker mothers. Maternal SEP factor (smaller value higher SEP) had a small negative indirect effect of −1.85 (−3.57 to −0.18) × 10^−3^ SD units (equal to −0.01 (−0.02 to −0.001) kg/m^2^) on BMI46 through 26 paths, as SEP improves (Fig. S6).

Birth weight was indirectly positively associated with BMI46, through 19 paths (Fig. S7) equal to 17.9 (5.5 to 29.3) × 10^−3^ SD units corresponding to 1.5 × 10^−4^ (4.5 × 10^−5^ to 2.4 × 10^−4^) kg/m^2^. In general, growth traits at infancy (1 y) and childhood (around 6 y) until adolescence (14 y) mediated the associations between prenatal variables and BMI46 (Fig. 2). Age at adiposity rebound (AgeAR), BMIAR11 and BMIAP had the largest, in absolute magnitude, indirect effects: AgeAR −174.0 (−219.0 to −124.3) ×10^−3^ i.e. increasing AgeAR associates with lower BMI46, BMIAR11 95.6 (39.1 to 158.0) ×10^−3^, BMIAP 53.1 (18.7–89.4) × 10^−3^ SD units. The corresponding values on their original scales are: AgeAR −0.99 (−1.24 to −0.71) kg/m^2^, which equals around 1 kg/m^2^ lower BMI46 by 11 months higher AgeAR, BMIAR11 2.19 (0.89–3.61) kg/m^2^ i.e. around 2.19 kg/m^2^ higher BMI46 by 0.21 kg/m^2^/year greater growth velocity between AgeAR and 11 y, BMIAP 0.31 (0.11–0.53) kg/m^2^ i.e. 0.3 kg/m^2^ higher BMI46 by around 0.8 kg/m^2^ higher BMIAP in infancy. AgeAR and BMIAR11 were associated with BMI46 through 2 paths (Figs S8 and S9, respectively, and one in each via blood pressure, BPF31) whilst BMIAP through 8 paths (Fig. S10). While BMI growth velocity between AR and 11 y mediated the association between early life factors and BMI46, the later growth speed (11–15 y) did not likely because of the impact of puberty.

The largest direct standardised effect on BMI46 was observed for the temporally adjacent phenotype of BMI31: 539.3 (500.3, 577.90) × 10^−3^ SD units corresponding to 0.68 (0.62, 0.72) kg/m^2^ on the original scale by 1 SD (~3.9 kg) increase in BMI31. BPF31 (factor score) had the fourth largest standardised effect after BMI31, AgeAR and BMIAR11: 80.4(46.1, 113.8) × 10^−3^ SD units equal to 0.05 (0.03 to 0.07) kg/m^2^ on the original scale.

**Fig. 3:**
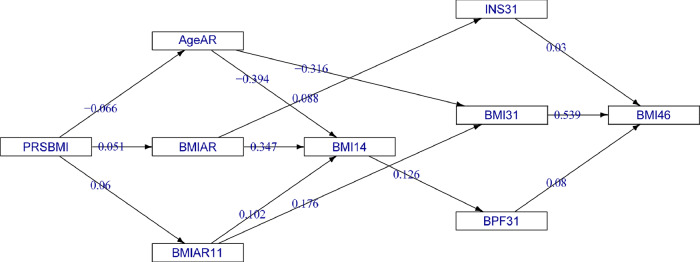
Subgraph of the directed acyclic graph (DAG) from the Bayesian path analysis model (BLSEM), showing all paths between PRSBMI and BMI46 thresholding for mean posterior probabilities (MPPIs) ≥ 0.5. The pairwise standardised effects (*β*s) × 10^3^ are presented on the edges (*n* = 4119). Direct effect = 0; total indirect effect = 14.8 (95% CI: 2.66, 22.8) SD units of BMI46 per 1-SD increase of PRSBMI; 6 pathways.

Genetic predisposition by PRSBMI had a moderate indirect effect on BMI46 equal to 14.8 (2.66, 22.8) × 10^−3^ SD units, suggesting that the genetic risk score may be capturing some of the genetic lifelong causes of BMI development. Importantly, the indirect associations through 6 paths showed that the genetic effect on adult BMI becomes apparent from the period of AR onwards. Figure 3 illustrates the subgraph of PRSBMI pathways for BMI46. The graph shows for example that increasing BMIPRS adversely decreases AgeAR and increases BMI velocity from AR to 11 y.

The percentage of variance in BMI explained by the fitted model, in terms of the Bayesian version of the *R*-squared, ranges for example from a posterior mean *R*^2^ of 14.7% for BMI at AP, up to 58.1% for BMI at 14 years (34.6% for BMI46). Other endogenous variables with high variance explained by the model include peak height velocity in infancy, PHV, with a posterior mean *R*^2^ of 34.8% and waist circumference with a posterior mean *R*^2^ of 24.0%. (Supplementary Table 4).

## Discussion

We have taken a systematic life-course approach to explore genetic and non-genetic factors associated with BMI development from the prenatal stage until middle age in a large population-based birth cohort study. The long follow-up, almost 50 years, of the study enabled us to investigate how early-life shapes, beginning with the intrauterine environment and continuing through infancy, childhood and puberty, the trajectory of body mass development throughout life.

One of the new observations is that the association of very early life factors with adult BMI is captured and mediated by growth patterns and other factors through childhood. We tested a large number of pre- and perinatal factors on BMI46 and all of them showed an indirect pattern, which is an important key finding. The subgraphs also illustrate the complexity of associations and help understand the mechanisms by which factors may work (e.g. Fig. 3). In previous studies, maternal BMI, maternal smoking, socio-economic status of the family and parity among others have been associated with BMI development, but the pathways through which they contribute have not been explored as in our study [37–39]. The analytical approaches may explain some variations between the associations in the studies, especially when a full life-course approach was not feasible.

In terms of the magnitude of the adult effect sizes, BMI31, the outcome of long-term development, is a dominating factor associated with BMI46 fifteen years later. This suggests that most people who have developed a high BMI by early adult life are likely to have a high BMI in middle age [13]. The AgeAR, usually occurring between ages 5 and 7 y, shows the second largest (inverse) standardised association with BMI46, indicating that the higher the age at AR, the lower the BMI46. Since an early AR reflects accelerated growth, the period by AgeAR is paramount for later BMI development and has potential for intervention. This is, for example, supported by earlier work of De Kroon et al., who show the mean age at AR in subjects with obesity was as early as 3 y, while for individuals without obesity it was 6 y [40].

We also newly discovered that the mean BMI growth velocity later in childhood, between the age at AR to 11 y has a strong independent association with BMI46 (on original scale 2.19 kg/m^2^ by 1 SD increase in growth velocity), with an additional path via blood pressure. This is supported by our earlier analyses on blood pressure, which showed that growth from AgeAR onwards had the largest estimated effect on blood pressure in young adulthood, promoting the view that early growth predicts well adult metabolic health [41].

Although other studies have highlighted the importance of early growth for later BMI development, the suggested critical time points have differed. In our analyses, BMIAP was also strongly associated with BMI46. Similar findings of strong positive effects of weight gain between the ages of 6 and 12 months and later obesity have been reported for other cohorts [15, 42–44]. Our results overall illustrate that adverse BMI development starts in early life, with growth parameters up to adolescence being good predictors of BMI until middle age, and the growth parameters themselves being affected by early environment. From an obesity development point of view, a key is to follow up child’s growth, to notify deviations from the expected measures and action.

We did not find a direct association of BW with later adiposity, but did find a relatively weak indirect association. This may be explained by a long timespan or the fact that the effects of BW are captured by other mediating factors across the life-course, which is also supported by a weak genetic correlation between BW and BMI later in life [17]. BW as a risk factor for obesity has been extensively studied with varying results, but this is the first time it has been studied within a life-course model simultaneously with a large number of potential contributors. In a review by Brisbois et al., 25 out of the 43 studies reported an association between BW and adult BMI [23]. Moreover, some studies show that low BW may predispose to higher BMI later in life, meaning that the association may not be linear. However, there was no indication of a U or J-shape association in our data.

Genetic predisposition captured by the PRS, BMIPRS, has a weak indirect positive association with BMI46 and only starts influencing BMI from the time of AR onwards. Likewise, we observed that the genetic factors influencing adult BMI were associated with BMIAR and AgeAR, but their overlap with BMIAP was either absent or weak [17]. Similar findings are supported by candidate gene and other smaller case studies [45, 46].

We conducted extensive sensitivity analyses to explore how the Bayesian Path Analysis Model, BLSEM, behaved under different scenarios i.e. fewer life stages or number of missing values. Given the reassuring results of these analyses, i.e. meaningful mean posterior regression coefficients and pathways, we feel that the BLSEM is a flexible tool for longitudinal analysis yielding valid results. An indication of successful selection of variables and successful model fitting was the fact that the model explained 35% of the variation at BMI46 and 58% for BMI14.

### Strengths and limitations

The major strength of this study is the life-course modelling approach, considering all measured potential contributors across the long timespan of the study. The Bayesian analytical method we developed provides a comprehensive way of jointly modelling all available information with no limit on the number of variables. Additionally, it yields easily interpretable estimates for both direct, indirect and total effects for each variable as well as uncertainty estimates on the regression coefficients and the DAG itself. Multiple missing values imputation is carried out simultaneously with model fitting, allowing to use the data to their full potential, thus maximising the statistical power of the study.

The main aim in choosing the comprehensive set of potential risk factors in the model is to include as many confounders and mediators as possible, in order to minimise the risk of unobserved confounding. Our principle is that we start with a large set of variables, which we hope will include all important risk factors and confounders, though it may also include unnecessary (‘noise’) variables as well. Our statistical model, however, performs automatic variable selection, which removes the noise variables and investigates how the influence of independent variables flows through multiple mediators before having an impact on the distal outcome.

We therefore start with an initial set of variables which includes all known risk factors that are available for our cohort, along with other potential risk factors and confounders which may have less well-established a priori evidence. The statistical model will detect which variables (potential risk factors and/or confounders) are needed to explain the variability in the endogenous variables. Therefore, our approach is both based on an a priori understanding of causal mechanisms and also uses a data-driven approach to select the final model.

Another strength is the long follow-up of NFBC1966 with a high attendance rate and the extensive growth data from birth until adolescence, offering opportunities to use complex models to obtain growth patterns more accurately [19, 21, 47].

Our study has certain limitations. The model requires the assumption of no unobserved covariates, relying on the breadth of measured potential covariates in NFBC1966. We used birth weight, taking into account gestational age as a surrogate measure of foetal growth, which does not fully capture intrauterine growth patterns. Moreover, BMI is a measure of total body mass and neither separates fat mass and fat-free mass nor accounts for body fat distribution. The choice of the above measures was made because of data availability and for consistency among the different life stages. We do not have enough information for adolescence, so we may have missed additional growth velocities related to later life BMI development. While the anthropometric data were directly measured, the velocity parameters were determined by the fitted growth curves, hence potentially resulting in less precise estimates of the associations between infant, childhood and pubertal growth with BMI46 than those between the BMI at each life stage. Life stages were defined by the data collection time points, forcing us to use 11 and 15 y as puberty cut points while other ages might have been more appropriate.

This study refers to a Finnish cohort and was initiated decades ago, meaning that results are likely to be generalisable to relatively high-income countries with similar characteristics and may not be directly applicable to today’s children. Across the life-course analyses always face this problem, having started half a century ago; however, they are vital to understand the bio-psycho-social interplay of different factors and to make inferences about the future prospects of the populations. From a methodological point, the method currently allows the analysis of continuous outcomes (dependent variables), but work is in progress to accommodate binary variables.

*In summary*, our method provides a flexible exploratory statistical tool with easily interpretable direct, indirect and total effect estimates for the analysis of longitudinal epidemiological data to formulate causal hypotheses for further investigation. This may not otherwise be possible using traditional analytical observational approaches [48, 49]. We acknowledge recently applied causal inference methods, such as Mendelian randomisation, and took our approach to assess all available data together, more than it has been possible to consider in the past, to get insights into possible mediating mechanisms. Our proposed approach, exploratory in nature, helps to get a better understanding of the pathways through which individuals may develop a clinical outcome later in life, taking into account a number of life-course determinants. Overall, this study illustrates that intervention measures may be planned as a long-term effort throughout childhood. However, further causal work on biological mechanisms and the best target periods is necessary. The model may be successfully adopted in other settings with fewer number of variables, less life stages or shorter follow-up periods. In our case study, the proposed method provides stable and consistent findings with previous observations, as far as we can compare those from less flexible and comprehensive analyses, suggesting that our methods yield meaningful and robust results.

## Supplementary information

BLSEM Supplementary material file25Rf2

## Data Availability

NFBC data are available from the University of Oulu, Infrastructure for Population Studies. Permission to use the data can be applied for research purposes via the electronic material request portal. In the use of data, we follow the EU General Data Protection Regulation (679/2016) and the Finnish Data Protection Act. The use of these data is based on the cohort participant’s written informed consent at his/her latest follow-up study, which may cause limitations to its use. Please, contact the NFBC project centre (NFBCprojectcenter@oulu.fi) and visit the cohort website (www.oulu.fi/nfbc) for more information. Model code is available as an open-source R package (BLSEM), incorporating a wrapper for the C++ Bayesian model estimation code, and several data and results visualisation functions written in R. The BLSEM package is publicly available at https://github.com/alexlewin24/BLSEM.
