# Supplementary material for: A Bayesian life-course linear structural equations model (BLSEM) to explore the development of body mass index (BMI) from the prenatal stage until middle age
Source: Int J Obes (Lond). 2025 Aug 20;49(10):2070–80. doi: 10.1038/s41366-025-01857-8 (PMC12532564; doi:10.1038/s41366-025-01857-8)
Supplement: Supplementary file 1 — BLSEM Supplementary material file25Rf2 [file 41366_2025_1857_MOESM1_ESM.pdf]

SUPPLEMENTARY MATERIAL

Table of Contents

Figure S1: Flowchart of main data collections and sample definition of the present study in NFBC1966 3

Figure S2: Geographic location of the NFBC1966. The dark grey shaded area indicates the catchment area of the cohort.......... 4

Table S1: Variable descriptions at each life stage detailing methods of collection and assessment (measurement), including original and modified coding of categorical variables. 5

Genotype data quality control 12

Model specification 12

Bayesian R squared.......... 12

Estimation algorithm and software.......... 13

Estimation of Causal Effects 13

Causal Assumptions.......... 14

Table S2: Descriptive statistics of the exogeneous (i.e. independent and explanatory) variables in the Northern Finland Birth Cohort 1966 Study Sample.......... 15

Figure S3: Correlation matrix between all endogenous and exogeneous variables in the study. 19

Table S3: Stage-specific results from the Bayesian path analysis model, BLSEM, with MPPIs≥ 0.5 for each endogenous variable (intermediate outcomes). Effect sizes (βs) are reported in SD units by SD or category change in exposure. All adjusted for sex. LCI= lower credible limit, UCI= upper credible limit. Variable and unit explanations in Table 1, Glossary and Table S1. 20

STAGE 1 MODEL.......... 20

STAGE 2 MODELS.......... 20

STAGE 3 MODELS.......... 21

STAGE 4 MODELS.......... 23

STAGE 5 MODELS.......... 24

STAGE 6 MODEL.......... 26

Table S4: Posterior mean estimates (%) and 95% CI of the Bayesian version of the R2 showing how much of the variation in each of the endogenous variable is explained by dependence on the exogenous variables.27

Model fitting procedure 27

Figure S4: Subgraph of the Directed Acyclic Graph (DAG) from the Bayesian Path Analysis Model, BLSEM, showing all paths thresholding for mean posterior probabilities (MPPIs) ≥0.5 between maternal BMI (matBMI) and BMI46. 28

Figure S5: Subgraph of the Directed Acyclic Graph (DAG) from the Bayesian Path Analysis Model, BLSEM, showing all paths thresholding for mean posterior probabilities (MPPIs) ≥0.5 between maternal smoking (matSMO) and BMI46. 29

Figure S6: Subgraph of the Directed Acyclic Graph (DAG) from the Bayesian Path Analysis Model, BLSEM, showing all paths thresholding for mean posterior probabilities (MPPIs) ≥0.5 between maternal SEP (matSEP) and BMI46. Better maternal socioeconomic position associated with lower middle-age BMI. 30

Figure S7: Subgraph of the Directed Acyclic Graph (DAG) from the Bayesian Path Analysis Model, BLSEM, showing all paths thresholding for mean posterior probabilities (MPPIs) ≥0.5 between birth weight (BW) and BMI-46. 31

Figure S8: Subgraph of the Directed Acyclic Graph (DAG) from the Bayesian Path Analysis Model, BLSEM, showing all paths thresholding for mean posterior probabilities (MPPIs) ≥0.5 between age at AR (AgeAR) and BMI46. 32

Figure S9: Subgraph of the Directed Acyclic Graph (DAG) from the Bayesian Path Analysis Model, BLSEM, showing all paths thresholding for mean posterior probabilities (MPPIs) ≥0.5 between Mean BMI velocity AR-11y (BMIAR11) and BMI46.......... 33

Figure S10: Subgraph of the Directed Acyclic Graph (DAG) from the Bayesian Path Analysis Model, BLSEM, showing all paths thresholding for mean posterior probabilities (MPPIs) ≥0.5 between BMI at AP (BMIAP) and BMI46. 34

References 36

Figure S1: Flowchart of main data collections and sample definition of the present study in NFBC1966 (1) **.**

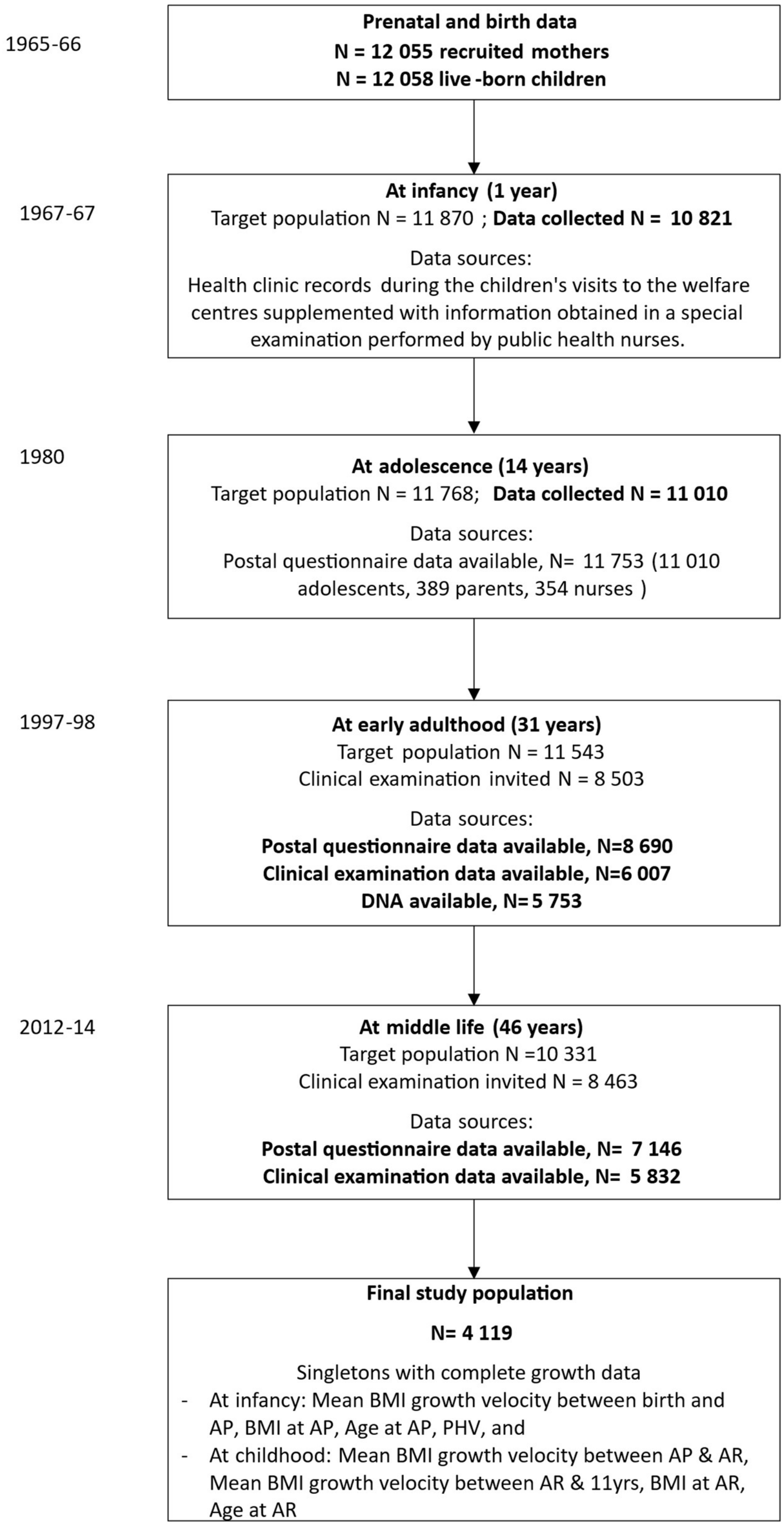

Abbreviations: NFBC1966: Northern Finland Birth Cohort 1966, BMI: body mass index, AP: adiposity peak; PHV: peak height velocity in infancy, AR: adiposity rebound.

Figure S2: Geographic location of the NFBC1966 (2) **. The dark grey shaded area indicates the catchment area of the cohort.**

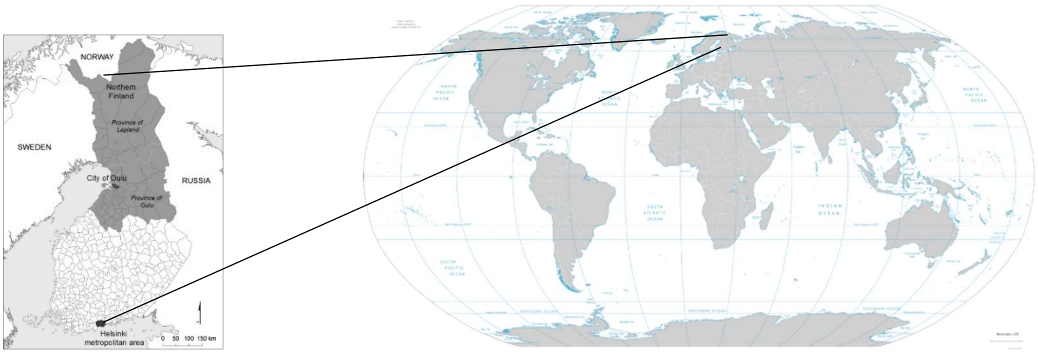

Table S1: Variable descriptions at each life stage detailing methods of collection and assessment (measurement), including original and modified coding of categorical variables.

Variable	Data collection method	Type of variable	Measurement methods
**Maternal**	Information on the mothers was collected by the local midwives in the antenatal clinics using questionnaire. The questionnaire was filled in from the 24th to 28th gestational week (i.e. women with pregnancy loss before 24th week excluded), but if this was not possible the questionnaire was completed later during the pregnancy or after the delivery (10.1% of mothers).		
Body Mass Index, kg/m^2	Questionnaire	Continuous	Height (bare footed) was measured in the maternity clinic and pre-pregnancy weight was self-reported. It was converted to BMI weight divided the squared height.
Age, years	Questionnaire	Continuous	Age was calculated from the date of birth of the mother at the child's birth.
Socioeconomic position (SEP) of the family	Derived (latent construct)	Continuous	As a measure of socio-economic position (SEP) for each individual, a factor score was created based on three variables: mother's and father's occupations categorized as 1=professional, 2=skilled worker/farmer, 3=unskilled worker, and a variable identifying farmer families. Smaller value indicated higher SEP.
Parity	Questionnaire	Discrete	Parity was defined as number of deliveries the mother has including this child.
Maternal marital status at birth	Questionnaire	Binary	Four response categories included married, unmarried, widowed, divorced. These were re-categorised for the data analysis into: 1: married, 0: otherwise
Maternal place of residence	Questionnaire	Categorical	Four response categories included city, small town, rural centre, remote village. These were re-categorised for the data analysis into 1: city, 2: small town & rural centre, 3: remote village.
Maternal smoking at the 2nd month of pregnancy	Questionnaire	Binary	Three response categories continued, stopped, did not smoke. These were re-categorised for the data analysis into 0: did not smoke, 1: continued & stopped smoking
Number of people in the household	Questionnaire	Discrete	Number of persons in the household (any persons, not only the family).
Combined 'wealth' indicator	Questionnaire	Ordinal	Calculated as the sum the three variables below with values 0, 1, 2 ,3:<br>a. Does the family's dwelling have telephone: no/yes<br>b. Does the family own a car: no/yes<br>c. Does the family own a summer cottage: no/yes
Maternal hypertension during pregnancy, mmHg	Clinical examination	Continuous	Maternal blood pressure (BP) measurements during pregnancy were obtained from health clinic records and were originally classified into nine categories:<br>1. **Gestational hypertension:** BP ≥ 145/95 mmHg after the 20th gestational week. Normal BP in the early pregnancy i.e. <20th gestational week. No proteinuria.

Variable	Data collection method	Type of variable	Measurement methods
			2. **Pre-eclampsia (PE):** BP as in 1 and with proteinuria at least in one sample during pregnancy.<br>3. **Chronic hypertension:** a. BP elevated already in early pregnancy (<20th gestational week), ≥ 145/95 mmHg and BP elevated during whole pregnancy or after pregnancy (6 weeks after pregnancy), b. Chronic hypertension (hypertension essentialis) diagnosis registered in questionnaire.<br>4. **Super-imposed pre-eclampsia:** BP as in 3 and proteinuria.<br>5. **Could not be determined:** All BP values not available.<br>6. **Not known:** all BP measurements during pregnancy missing<br>7. **Systolic BP elevated:** - Systolic BP ≥ 145 mmHg and diastolic BP < 95 mmHg, must be analysed separately, includes women with proteinuria.<br>8. **Normotensive:** BP < 145/95 mmHg during the whole pregnancy and is not included in any of the above-mentioned groups.<br>9. **Diastolic BP elevated:** - Diastolic BP ≥ 95 mmHg and systolic BP < 145 mmHg, must be analysed separately, includes women with proteinuria.<br>These were re-categorised into seven groups collapsing 2 & 4 in one group and 5 & 6 in another group. Normotensive was taken as the reference category.
Paternal age, years	Questionnaire	Continuous	Age of the father at child's birth.
**At birth**	Information on the offspring at birth were obtained from clinic records.		
Polygenic risk score for BMI	From child's genome-wide genotyped data (sampling at 31 years)	Continuous	The computation is described below.
Sex of the offspring	Clinical examination	Binary	1: boy 0: girl
Birth weight, kg	Clinical examination	Continuous	Birthweight (± 5g) of the offspring was measured by nurse immediately after birth.
Gestational age, weeks	Derived	Continuous	Calculated time between date of birth of the child and last menstrual period of the mother.

Variable	Data collection method	Type of variable	Measurement methods
Operative managements in delivery	After delivery from records	Binary	Originally grouped to four categories: nothing, caesarean section, vacuum extraction or forceps, breech delivery. Dichotomised for this analysis as 1: caesarean section 0: otherwise.
Placenta weight, grams	Clinical examination	Continuous	Measured after delivery by attending nurse.
**At infancy – 1y**	Postnatal height and weight growth were obtained from child health records stored in municipal health centres (child welfare centres). All children had multiple (on average 20) height and weight measurements from birth until adolescence, with more frequent measurements during the first year of life and approximately one measurement per year thereafter. The BMI growth pattern is nonlinear as part of the normal growth and development of a child. Two periods were considered: infancy from 2 weeks to 18 months and childhood from 18 months to 13 years in growth modelling. Age and BMI at adiposity peak (AP) and rebound (AR) were derived from random effects models from the two age groups separately as described previously (3–5).		
BMI at AP, kg/m^2	Derived from growth curves	Continuous	In infancy, BMI increases from birth, reaching a so-called infancy peak BMI at approximately 9 months of age.
Age at AP, years	Derived	Discrete	Defined as the age at which BMI peak occurs (inflexion point) on the growth curve and is usually between the ages of 0.25 to 1.25 years.
Mean BMI growth velocity between birth & AP, (kg/m^2)/year	Derived from growth curves	Continuous	Growth charts and modelling.
Peak height velocity cm/year	Derived from growth curves	Continuous	Growth charts and modelling.
**At childhood – 6y**			
BMI at AR, kg/m^2	Derived from growth curves	Continuous	Growth charts and modelling.
Age at AR, years	Derived from growth curves	Discrete	Defined as the age at which BMI nadir occurs (inflexion point) on the growth curve and ranges between the ages of 2.5 to 8.25 years in different populations.
Mean BMI growth velocity between AP & AR, (kg/m^2)/year	Derived from growth curves	Continuous	Growth charts and modelling.
Mean BMI growth velocity between AR & 11years, (kg/m^2)/year	Derived from growth curves	Continuous	Growth charts and modelling.

Variable	Data collection method	Type of variable	Measurement methods
**At adolescence – 14y**		Information concerning growth and health, living habits, school performance and family conditions of all adolescents alive at 14 years of age were obtained through postal questionnaires. Postal questionnaire was sent to the adolescents but if they did not respond then to the parents. If neither of them responded, then data were retrieved from school welfare clinics.	
BMI at 14 years, kg/m2	Derived from growth curves	Continuous	Height and weight measured in the clinical examination at school welfare clinics that the participants attended to an accuracy of 0.1 cm and 0.1 kg, respectively and converted to BMI (kg/m^2).
Mean BMI velocity 11-15 years, (kg/m^2)/year	Derived from growth curves	Continuous	Mean growth velocity for BMI between the age of 11 and 15 years (remodelled until 15y).
Socioeconomic position (SEP) of the family	Derived (latent construct)	Categorical	As a measure of socio-economic position (SEP) for each individual, a factor score was created based on three variables: mother's and father's occupations categorized as 1=professional, 2=skilled worker/farmer, 3=unskilled worker, and a variable identifying farmer families. Smaller value indicated higher SEP.
Smoking	Questionnaire	Binary	Originally grouped to eight categories: never tried, tried once, tried a few times, smoke occasionally, smoke about twice a week, smoke 1-5 cigarettes/day, smoke 6-10 cigarettes/day, smoke >10 /day. Dichotomised for this analysis as 'non-smoker' [never tried, tried once, tried a few times],'occasional/regular smoker' otherwise.
Alcohol use	Questionnaire	Binary	Originally grouped to five categories: never drunk any, tasted once, drunk a few times, use alcohol monthly, use alcohol weekly. Dichotomised for this analysis as 'non-consumer' [never drunk any, tasted once], 'regular consumer' otherwise (6) .
Physical activity	Questionnaire	Binary	Evaluated with question on the frequency of doing sports after school hours. Originally grouped to seven categories: every day, every second day, twice a week, once a week, every second week, once a month, usually never. Dichotomised for this analysis as 'less than once a week' (every second week, once a month, usually never) and 'once a week or more' otherwise (7) .
**At early adulthood – 31y**		Information concerning basic characteristics, family, occupation, living environment, health, eating and behavioural habits were obtained through postal questionnaires to the participants at 31 years. Clinical examinations were carried out at this age.	
Body Mass Index (kg/m^2)	Clinical examination	Continuous	Height and weight measured in the clinical examination that the participants were invited to an accuracy of 0.1 cm and 0.1 kg, respectively and converted to BMI (kg/m^2).
Socioeconomic position (SEP)-Factor 1	Derived (latent construct)	Continuous	As a measure for SEP, factor score for each individual based on confirmatory factor analysis and represented by basic education (0.709), further education (0.483), occupation (0.644)

Variable	Data collection method	Type of variable	Measurement methods
			and household income (0.481) at 31 years of age. Numbers in parenthesis are the factor loadings (8) .
Socioeconomic position (SEP)-Factor 2	Derived (latent construct)	Continuous	As a measure of SEP, factor score for each individual was created based on three variables: occupational level (1=upper-level employees, 2=lower-level employees/entrepreneurs, 3=manual workers/farmers, 4= not working) and identification variables for entrepreneurs and farmers. Smaller value indicated higher SEP.
Number of adults living in the household	Questionnaire – self reported	Discrete	Number of persons in the individual's family or household including themselves.
Number of children living in the household	Questionnaire – self reported	Discrete	Number of children (aged under 18 years) in the individual's family or household.
Smoking	Questionnaire – self reported	Binary	Based on two questions asking whether participants have ever smoked in their life and whether they smoke nowadays. Originally grouped to three categories: non-smoker, occasional, regular smoker (9) . For this analysis dichotomised to 'non-smoker', 'occasional/regular smoker).
Smoking pack years	Questionnaire – self reported	Continuous	Calculated as the number of cigarettes smoked /day divided by 20 and multiplied by the number of years smoked, for current smokers or for those that smoked during the last 12 months. Others were asked to skip the relevant question on the number of cigarettes smoked /day. Note: 1 cigar = 5 cigarettes.
Alcohol use	Questionnaire – self reported	Continuous	Assessed with several questions on the type and amount of the consumed alcohol and transformed into grams /day (10).
Diet score	Questionnaire – self reported	Continuous	Consumption of food and beverages were surveyed using a 32-item food frequency questionnaire depicting the participants' habitual food consumption during the previous 6 months. Items describing healthy diet were frequent consumption of plain dairy yogurts, rye bread/crispbread, porridge, salad dressings, fresh vegetables, cooked vegetables, fruits, fresh or frozen berries and fish (9 food items). Items describing unhealthy diet were frequent consumption of sausages/frankfurters, cold cuts, fried potatoes/French fries, sugar-sweetened soft drinks, white bread, and hamburgers and pizzas (6 food or drink items). On each item, either zero (less frequent consumption) or one point (more frequent consumption) was assigned and sum scores for healthy and unhealthy diets were calculated. Scores for unhealthy and healthy diet were then factorized into one variable (11); the smaller the value the better.
Physical activity, MET-hours/week	Questionnaire – self reported	Continuous	Leisure time physical activity was evaluated with questions on the frequency and duration of light and brisk physical activities during leisure time. The data were transferred into

Variable	Data collection method	Type of variable	Measurement methods
			metabolic equivalent (MET) hours/ week and using the intensity values of 3 and 5 METs for light and brisk physical activities, respectively. The data were then classified according to the sex-specific quartile limits (males: 3.0, 9.0, and 20.0 MET hours /week; females 4.8, 11.6 and 21.0 MET hours/week) (7) .
Blood pressure latent factor	Derived (latent construct)	Continuous	Factor scores based on confirmatory factor analysis and represented by systolic (0.802) and diastolic (0.794) blood pressure at 31 years of age. Numbers in parenthesis are the factor loadings (8) .
Psycho-social latent factor	Derived (latent construct)	Continuous	Factor scores based on confirmatory factor analysis and represented by marital status (0.427), employment status (0.344), home ownership (0.362), depression (0.684), sleep (0.556) and quality and life satisfaction (0.642) at 31 years of age. Numbers in parenthesis are the factor loadings (8) .
Insulin, µIU/mL	Clinical examination	Continuous	Fasting serum insulin levels were determined by radioimmunoassay (Pharmacia Diagnostics, Uppsala, Sweden).
Waist circumference, cm	Clinical examination	Continuous	Measured from the point midway between the costal margin and iliac crest and recorded to an accuracy of 0.1cm.
High Density Lipoproteins Cholesterol (HDL-C), mmol/L	Clinical examination	Continuous	Analysed using a Hitachi 911 Chemistry Analyser and commercial reagents (Boehringer Mannheim, Germany).
Low Density Lipoproteins Cholesterol (LDL-C), mmol/L	Clinical examination	Continuous	Friedewald equation: LDL-C= (Total Cholesterol) – (HDL-C) – (TGs/5).
Triglycerides (TG), mmol/L	Clinical examination	Continuous	Analysed using a Hitachi 911 Chemistry Analyser and commercial reagents (Boehringer Mannheim, Germany).
**At late adulthood – 46y**			
Body Mass Index (kg/m^2)	Clinical examination	Continuous	Height and weight measured in the clinical examination that the participants were invited to an accuracy of 0.1 cm and 0.1 kg, respectively and converted to BMI (kg/m^2).
Socioeconomic position (SEP)	Derived (latent construct)	Continuous	As a measure of SEP, a factor score for each individual was created based on three variables: occupational level (1=upper-level employees, 2=lower-level employees/entrepreneurs, 3=manual workers/farmers, 4= not working) and identification variables for entrepreneurs and farmers. Smaller value indicated higher SEP.

Variable	Data collection method	Type of variable	Measurement methods
Smoking	Questionnaire – self reported	Binary	Based on two questions asking whether participants have ever smoked in their life and whether they smoke nowadays. Originally grouped to three categories: non-smoker, occasional, regular smoker (9) . For this analysis dichotomised to 'non-smoker', 'occasional/regular smoker'.
Alcohol consumption	Questionnaire – self reported	Continuous	Assessed with several questions on the type, amount and frequency of alcohol consumption and the information was validated against 7-day food diaries. Then, it was transformed into daily intake (g/day) (12) .
Diet score	Derived (latent construct)	Continuous	Consumption of food and beverages were surveyed using a 32-item food frequency questionnaire depicting the participants' habitual food consumption during the previous 6 months. Items describing healthy diet were frequent consumption of plain dairy yogurts, rye bread/crispbread, porridge, salad dressings, fresh vegetables, cooked vegetables, fruits, fresh or frozen berries and fish (9 food items). Items describing unhealthy diet were frequent consumption of sausages/frankfurters, cold cuts, fried potatoes/French fries, sugar-sweetened soft drinks, white bread, and hamburgers and pizzas (6 food or drink items). On each item, either zero (less frequent consumption) or one point (more frequent consumption) was assigned and sum scores for healthy and unhealthy diets were calculated. Scores for unhealthy and healthy diet were then factorized into one variable (11) ; the higher the value the better.

Abbreviations: NFBC1966: Northern Finland Birth Cohort 1966.
Note: Information taken from the NFBC1966 (https://www.oulu.fi/nfbc/1966datacollections) and from specific publications as shown in the Table.

Genotype data quality control

Genotyping of the NFBC1966 participants was carried out at the Broad Institute, USA, using the Illumina Infinium 370cnvDuo array and the Beadstudio calling algorithm, as described previously by Sabatti et al (13) . Individuals with low call rate (<95%), unspecified sex, sample duplication/contamination, sex mismatch, relatedness (identity by descent [IBD]), outlying heterozygosity or withdrawal of consent, were excluded giving an initial sample size of 5400. Population stratification was assessed by multidimensional scaling analysis (MDS) and compared with Hapmap phase 3 reference populations; no individuals of non-European ancestry were detected. Copy number variations (CNVs) and single nucleotide polymorphisms (SNPs) with call rate <95% (for markers with minor allele frequency [MAF] >5%), CNVs and SNPs with call rate <99% (for markers with MAF <5%), lack of Hardy-Weinberg equilibrium (HWE) (P <1.0 x 10-4) or MAF <1% were excluded (leaving 4856 subjects). Array genotypes were harmonised and imputed to the Haplotype Reference Consortium (HRC) imputation reference panel (14) via the Michigan imputation server (15). We excluded imputed SNPs with minor allele count (MAC) <5, imputation quality score (r2) <0.3 or evidence for Hardy-Weinberg disequilibrium (P <1e-6); we included only autosomal SNPs and used hard called genotypes (as output by the minimac3 software package (3)) in subsequent analyses.

Model specification

Label blocks $q = 1,\cdots, Q$, and variables within block q by $k = 1,\cdots, K_q$. The Bayesian framework requires specification of the likelihood and priors on all parameters. The likelihood for the path analysis model is the product of regression models for all endogenous variables from all blocks. For response variable *k* of block *q*, the regression model equation can be written

$$y_{qk} = Z_q\alpha_{qk} + X_q\beta_{qk} + Y_q\lambda_{qk} + \varepsilon_{qk}$$

where the y_{qk} is the vector of all observations of the response variable. The matrix Z_q is the design matrix of all variables in the parent blocks of block q that are to be forced in the regression. Likewise, X_q and Y_q are respectively the matrices of the exogenous and endogenous variables from the parent blocks. The vectors α_{qk}, β_{qk} and λ_{qk} are the regression coefficients corresponding to the three sets of covariates, and ε_{qk} are the residuals, assumed to follow the Normal distribution with variance σ_{qk}^2. The coefficients for variables always in the regression are given vague priors: $\alpha_{qk} \sim N(0, \sigma_\alpha^2)$, and the residual variances have $(\sigma_{qk})^{-2} \sim \text{Gam}(a_\sigma, b_\sigma)$.

Bayesian variable selection (BVS) spike and slab priors are used for the β_{qk} coefficients:

$$\beta_{qkj}|\gamma_{qkj} \sim \gamma_{qkj}N(0,\sigma_\beta^2) + (1-\gamma_{qkj})\delta_0$$
$$\gamma_{qkj} \mid \omega_{qj} \sim Bern(\omega_{qj})$$
$$\omega_{qj} \sim Beta(a_\omega, b_\omega)$$

where γ_{qkj} is an indicator variable for covariate *j* in matrix X_q to be selected into the regression model for response *q, k*. The δ_0 stands for the delta-function, which sets the β_{qkj} to zero when γ_{qkj} is zero. A similar BVS prior is used for the λ_{qk} parameters.

The hyperparameters a_ω, b_ω, σ_β^2, σ_α^2, a_σ, b_σ are fixed to values giving wide priors for the hierarchical parameters. For this study, 19 simultaneous equations were fitted with birth weight, BMI, growth parameters and metabolic traits as the outcome variables depending on the life-stage (blue boxes in Figure 1 in the main document).

Bayesian R squared

In order to see how much of the variation in each endogenous variable is explained by dependence on the exogenous variables, we obtain a Bayesian version of R squared for each endogenous variable (16). In an analogous way to the frequentist version, the Bayesian R squared for variable *k* in block *q* is defined as

$$R^2_{kq} = \frac{var^{fit}_{kq}}{var^{fit}_{kq} + var^{res}_{kq}}$$

where var^{fit}_{kq} is the sample variance (over the *n* individuals) of the fitted values $Z_q\alpha_{qk} + X_q\beta_{qk} + Y_q\lambda_{qk}$ and var^{res}_{kq} is the model residual variance (here σ^2_{qk}). We obtain the whole posterior distribution of R^2_{kq} for each endogenous variable. The uncertainty in the posterior distribution includes uncertainty from imputation as well as the uncertainty in the model coefficients.

Estimation algorithm and software

The model is estimated using Markov Chain Monte Carlo (MCMC), using Metropolis-Hastings and reversible jump moves for updating model parameters. Since the space of all possible regression models is so large (each regression equation has 2^{pq} possible configurations where *p* and *q* are the total numbers of variables in X_q and Y_q respectively), multiple parallel chains are run using local and global update moves, based on the ESS sampler (17). The software is available as an R package in https://github.com/alexlewin24/BLSEM.

Estimation of Causal Effects

Our aim is to model the development of BMI through several life stages as a function of a large number of exposures and potential mediating factors. A link can be made between our model estimates and causal estimands, under certain assumptions.

In order to define causal estimands, we work in the counterfactual potential outcomes' framework. The potential outcome for an individual is defined as the outcome that individual would have given certain values of the exposures and mediators. Since one individual only experiences one value of each of the exposures and mediators, only one potential outcome is observed for each individual, the rest remaining counterfactual. Missing (unobserved) potential outcomes can be estimated by assuming exchangeability of individuals with similar values of exposures and mediators.

Causal mediation estimands can also be estimated if certain assumptions are made. We estimate Natural Direct Effects (NDEs) and Natural Indirect Effects (NIEs) for each exposure variable of interest. To define these, we first have to define potential values of the mediators and outcome. For a given exposure variable, define M(a) to be the vector of potential values of the mediators an individual would experience given that they had exposure value a. The potential outcome Y(a, M(a*)) is defined as the outcome an individual would have given that their exposure took value a but their mediators took the natural values M(a*) where a* may be a different value of the exposure. The natural direct effect NDE(a) is defined as E[Y(a, M(a)) - Y(a*, M(a))], i.e. the difference in outcomes for different exposure values with the mediators held constant at the values they would take under exposure value a. The natural indirect effect NIE(a) is E[Y(a, M(a)) - Y(a, M(a*))], the difference in outcomes when the exposure is held constant but the mediators change. This is the effect of the exposure mediated through all intermediate variables. The Total Effect of the exposure is E[Y(a, M(a)) - Y(a*, M(a*))], the difference in outcomes when all mediators take their natural values under a vs a*. If we make the assumption that NDE(a) and NIE(a) are the same for all values of the exposure a, then the Total Effect can be decomposed into the sum of the NDE and the NIE.

We use linear structural equation models (LSEM) to model the relations amongst variables. In an LSEM with no interaction terms, the natural direct and indirect effects defined above can be expressed in terms of path-specific effects (18). Path specific effects are estimated as the products of regression coefficients over a given

pathway in the DAG. In our work we do not interpret each path specific effect separately, but instead we sum over all indirect paths to obtain an overall estimate of the natural indirect effect of a particular exposure on the BMI outcome (mediated through all the intermediate variables included in the model).

We have developed a Bayesian version of an LSEM (BLSEM) adapted for large numbers of variables, using variable selection priors to find a subset of the mediators and covariates to include in the regression model. The model allows for uncertainty about which covariates and mediators should enter into each component regression model. Bayesian model averaging is used to produce estimates of regression coefficients with this uncertainty included. We also obtain posterior probabilities for each variable to be included in each regression model. This can provide further information and evidence about which are the most important mediators. Variable selection and regularisation of coefficients in regression models have long been known to give increased power to detect important variables in regression models in comparison to unregularised models (19). Bayesian model averaging further provides a formal way to incorporate model uncertainty into inference. Recently two Bayesian LSEM with regularisation have been used to estimate causal effects with high-dimensional mediators (20,21). Our model takes a similar approach to that of Song et al. 2020, estimating a Bayesian LSEM with regularisation priors and Bayesian model averaging.

Causal Assumptions

In order to estimate the natural direct and indirect effects, we make the standard assumption of sequential ignorability: this means that we assume (i) there is no unmeasured confounding of the effect of the exposure on either mediators or outcome, and (ii) that there is no unmeasured confounding of the effect of the mediators on the outcome, conditional on the exposure.

Taking the genetic risk score PRS as exposure of interest, the first assumption seems broadly reasonable, since we should not expect shared causal factors for PRS and later phenotypes. The second is more difficult, as we cannot guarantee to have recorded all confounders of later growth and lifestyle variables. However, our study has the advantage that we are able to include a large number of observed confounders in the analysis, thus greatly reducing the effect of confounding in comparison to smaller studies.

We must also make the assumption that the potential outcomes of mediators and outcome variables are independent conditional on the exposure. This assumption effectively means that there are no additional mediators which are causally related to the observed mediating variables: any other mediating variables must act on separate pathways to those that are included in the study. This is also difficult to guarantee.

However again we have an advantage over studies considering one mediator at a time: since we consider many mediating variables we are more likely to include the majority of causally related pathways. This advantage of using multiple mediators is discussed in Huang and Pan (22) and VanderWeele and Vansteelandt (18) .

We make the stable unit treatment value assumption (SUTVA) which is standard in causal analysis, and we need the assumption of temporal ordering, which we have built into the model DAG. In order for our estimates to be reasonable, we also rely on the assumption of linearity in the path analysis model. We have taken care to transform variables where appropriate so that the linearity assumption is reasonable.

The final assumption that we have employed is that of no exposure-mediator interactions in the linear models, which means that we can estimate the causal effects as sums of path specific effects. This assumption is the most difficult to address in our work. We have employed this assumption so that we can explore predictors of

growth of the life course, including a large number of variables in the model. Further work should be done to explore in more detail possible interactions between mediators and exposures.

Table S2: Descriptive statistics of the exogeneous (i.e. independent and explanatory) variables in the Northern Finland Birth Cohort 1966 Study Sample.

Characteristics	Males (N=up to 2154)	Females (N=up to 1965)	Total (N=up to 4119)
**Maternal/Prenatal characteristics**			
**Maternal BMI (kg/m²)**			
N	1972	1819	3791
Mean (SD)	23.0 (3.1)	23.0 (3.2)	23.0 (3.1)
**Maternal age (years)**			
N	2154	1965	4119
Mean (SD)	27.2 (6.4)	27.1 (6.3)	27.1 (6.4)
**SEP of the family (factor score)**			
N	2154	1965	4119
Median (Q1, Q3)	-0.1 (-1.1, 0.7)	0.1 (-1.0, 0.7)	0.1 (-1.0, 0.7)
**Parity**			
N	2149	1963	4112
Median (Q1, Q3)	2.0 (1.0, 3.0)	2.0 (1.0, 3.0)	2.0 (1.0, 3.0)
**Maternal marital status at birth**			
Unmarried, divorced or widowed	68 (3.2%)	68 (3.5%)	136 (3.3%)
Married	2085 (96.8%)	1891 (96.5%)	3976 (96.7%)
**Maternal place of residence**			
City	706 (32.8%)	721 (36.7%)	1427 (34.6%)
Small town or rural centre	725 (33.7%)	624 (31.8%)	1349 (32.8%)
Remote village	723 (33.6%)	620 (31.6%)	1343 (32.6%)
**Maternal smoking**			
Never	1646 (78.0%)	1532 (79.5%)	3178 (78.8%)
Ever	463 (22.0%)	394 (20.5%)	857 (21.2%)
**Number of people in the house**			
N	2128	1921	4049
Median (Q1, Q3)	3.0 (2.0, 5.0)	4.0 (2.0, 5.0)	4.0 (2.0, 5.0)
**Combined wealth indicator**			
N	1948	1765	3713
Median (Q1, Q3)	1.0 (0.0, 2.0)	1.0 (0.0, 2.0)	1.0 (0.0, 2.0)
**Maternal hypertension during pregnancy**			

**(based on blood pressure, mmHg)**			
Normotensive	1216 (57.1%)	1107 (57.0%)	2323(57.0%)
Gestational hypertension	185 (8.7%)	179 (9.2%)	364 (8.9%)
Chronic hypertension	119 (5.6%)	112 (5.8%)	231 (5.7%)
Pre-eclampsia & Super-imposed pre-eclampsia	70 (3.3%)	64 (3.3%)	134 (3.3%)
Systolic blood pressure elevated	176 (8.3%)	158 (8.1%)	334 (8.2%)
Diastolic blood pressure elevated	171 (8.0%)	155 (8.0%)	326 (8.0%)
Not determined/Not known	194 (9.1%)	167 (8.6%)	361 (8.9%)
**Paternal age (years)**			
N	2052	1876	3928
Mean (SD)	30.4 (6.9)	30.5 (6.9)	30.5 (6.9)
**Characteristics at birth**			
**BMI PRS**			
N	1117	1161	2278
Mean (SD)	$2.61 X10^{-7}$ ($5.52 X10^{-7}$)	$2.91 X10^{-7}$ ($5.66 X10^{-7}$)	$2.77 X10^{-7}$ ($5.60 X10^{-7}$)
**Gestational age (weeks)**			
N	2097	1913	4010
Median (Q1, Q3)	40.0 (39.0, 41.0)	40.0 (39.0, 41.0)	40.0 (39.0, 41.0)
**Operative management in delivery**			
Vaginal	736 (86.9%)	656 (89.0%)	1392 (87.9%)
Caesarian section	111 (13.1%)	81 (11.0%)	192 (12.1%)
**Placental weight (grams)**			
N	1884	1742	3626
Mean (SD)	657.1 (143.7)	642.8 (138.3)	650.2 (141.3)
**Characteristics at 14y**			
**Smoking at 14y**			
Non-smoker	1720 (85.0%)	1553 (81.9%)	3273 (83.5%)
Occasional/regular smoker	303 (15.0%)	343 (18.1%)	646 (16.5%)
**Alcohol use at 14y**			
Non-consumer	1572 (77.6%)	1400 (73.9%)	2972 (75.8%)
Regular consumer	453 (22.4%)	494 (26.1%)	947 (24.2%)
**Physical activity at 14y**			
More than once a week	1657 (83.1%)	1286 (68.7%)	2943 (76.1%)
Less than once a week	337 (16.9%)	585 (31.3%)	922 (23.9%)

**SEP of the family at 14y (factor score)**			
N	2154	1965	4119
Median (Q1, Q3)	-0.2 (-1.0, 0.6)	-0.2 (-1.0, 0.6)	-0.2 (-1.0, 0.6)
**Characteristics at 31y**			
**Smoking at 31y**			
Non-smoker	814 (51.2%)	1026 (63.6%)	1840 (57.4%)
Occasional/regular smoker	777 (48.8%)	587 (36.4%)	1364 (42.6%)
**Smoking pack years at 31y**			
N	1005	931	1936
Median (Q1, Q3)	3.2 (0.0, 12.0)	0.0 (0.0, 6.5)	1.0 (0.0, 9.0)
**Alcohol use at 31y (g/day)**			
N	1562	1572	3134
Median (Q1, Q3)	8.7 (3.1, 18.8)	2.3 (0.6, 6.2)	4.6 (1.2, 11.8)
**Diet score at 31y (factor score)**			
N	1514	1552	3066
Mean (SD)	0.4 (1.0)	-0.4 (0.9)	0.0 (1.0)
**Physical activity at 31y (MET-hours/week)**			
N	1555	1580	3135
Median (Q1, Q3)	8.8 (2.7, 17.1)	10.1(4.3, 16.9)	9.4 (3.5, 16.9)
**Number of adults living in the household at 31y**			
N	1531	1590	3121
Median (Q1, Q3)	2.0 (2.0, 2.0)	2.0 (2.0, 2.0)	2.0 (2.0, 2.0)
**Number of children living in the household at 31y**			
N	1510	1581	3091
Median (Q1, Q3)	1.0 (0.0, 2.0)	1.0 (0.0, 2.0)	1.0 (0.0, 2.0)
**SEP latent factor-1 at 31y**			
N	921	1119	2040
Median (Q1, Q3)	0.2 (-0.3, 0.5)	-0.1 (-0.5, 0.2)	0.0 (-0.4, 0.4)
**SEP latent factor-2 at 31y**			
N	1394	1353	2747
Median (Q1, Q3)	-0.2 (-0.7, 0.5)	0.2 (-0.6, 0.7)	-0.0 (-0.7, 0.6)
**Psycho-social latent factor at 31y**			
N	921	1119	2040
Median (Q1, Q3)	-0.1 (-0.3, 0.1)	-0.0 (-0.2, 0.2)	-0.1 (-0.2, 0.2)
**Characteristics at 46y**			

**SEP latent factor at 46y**			
N	1122	1202	2324
Median (Q1, Q3)	-0.2 (-0.8, 0.8)	-0.0 (-0.6, 0.8)	-0.1 (-0.8, 0.8)
**Smoking at 46y**			
Non-smoker	471 (37.5%)	645 (45.8%)	1116 (41.9%)
Occasional/regular smoker	785 (62.5%)	762 (54.2%)	1547 (58.1%)
**Alcohol use at 46y (g/day)**			
N	1262	1410	2672
Median (Q1, Q3)	8.7 (2.4, 21.9)	3.0 (0.6, 8.5)	5.0 (1.1, 14.9)
**Diet score at 46y (factor score)**			
N	976	1131	2107
Mean (SD)	-0.4 (1.0)	0.3 (0.9)	-0.0 (1.0)

Note: Data are reported as mean (SD) for normally distributed intervals, median (interquartile range (IQR)) for non-parametrically distributed continuous variables and n (%) for categorical variables.

Figure S3: Correlation matrix between all endogenous and exogeneous variables in the study.

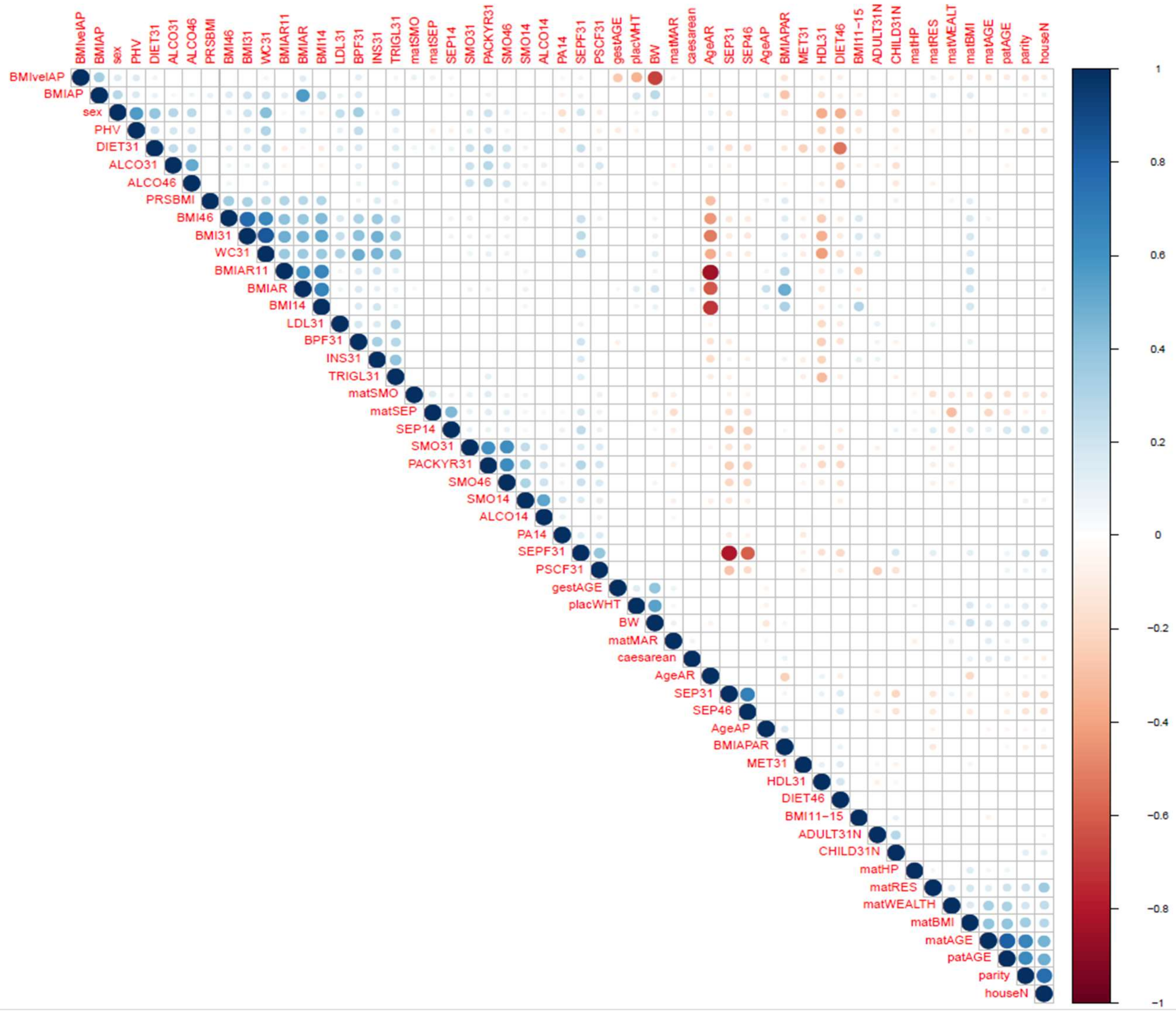

Table S3: Stage-specific results from the Bayesian path analysis model, BLSEM, with MPPIs≥ 0.5 for each endogenous variable (intermediate outcomes). Effect sizes (βs) are reported in SD units by SD or category change in exposure. All adjusted for sex. LCI= lower credible limit, UCI= upper credible limit. Variable and unit explanations in Table 1, Glossary and Table S1.

STAGE 1 MODEL

Stage 1 model, outcome variable: Birth Weight (BW)

Model		Mean pp (γ)	Direct effect β (x10 $^{-3}$)	95% LCI	95% UCI
**Prenatal**					
Maternal BMI		1.00	74.98	49.90	99.60
Maternal age		1.00	26.87	-2.50	55.60
SEP of the family		1.00	-48.56	-72.50	-25.00
Maternal place of residence					
City	Referent				
Remote village		1.00	-108.15	-159.40	-55.90
Maternal smoking					
Non-smoker	Referent				
Continued/stopped		1.00	-185.36	-241.80	-128.80
Number of people in the household		1.00	66.52	37.40	96.40
Maternal hypertension					
Normotensive	Referent				
Chronic hypertension		0.94	-81.05	-183.50	12.90
Pre-eclampsia (PE) & Super-imposed PE		1.00	-407.65	-531.70	-280.80
**At birth**					
Gestational age		1.00	314.98	286.90	342.50
Placenta weight		1.00	318.64	292.20	344.70

STAGE 2 MODELS

Stage 2 model, outcome variable: mean BMI velocity birth – adiposity peak (BMIvelAP)

Model		Mean pp (γ)	Direct effect β (x10 $^{-3}$)	95% LCI	95% UCI
**Prenatal**					
Maternal place of residence					
City	Referent				
Small town & rural centre		0.94	102.53	0.00	152.30
Remote village		1.00	159.47	97.10	216.60
**At birth**					
BMI PRS		0.01	0.03	0.00	0.00
Birth weight		1.00	-796.59	-822.10	-771.50

Stage 2 model, outcome variable: BMI at adiposity peak (BMIAP)

Model		Mean pp (γ)	Direct effect β (x10^{-3})	95% LCI	95% UCI
**Prenatal**					
Maternal BMI		0.95	55.84	0.00	83.70
Maternal place of residence					
City	Referent				
Small town & rural centre		0.84	112.34	0.00	189.20
Remote village		0.99	184.79	94.10	270.90
Maternal smoking					
Non-smoker	Referent				
Continued/stopped		0.99	167.55	94.40	245.30
**At birth**					
Gestational age		0.84	-57.88	-96.70	0.00
Birth weight		1.00	292.39	250.20	331.90

Stage 2 model, outcome variable: Age at adiposity peak (AgeAP)

Model		Mean pp (γ)	Direct effect β (x10^{-3})	95% LCI	95% UCI
Birth weight		1.00	-143.01	-177.70	-107.10

Stage 2 model, outcome variable: Peak height velocity (PHV)

Model		Mean pp (γ)	Direct effect β (x10^{-3})	95% LCI	95% UCI
**Prenatal**					
Maternal age (years)		0.53	34.62	0.00	90.10
SEP of the family		0.76	-40.82	-74.30	0.00
Parity		1.00	-162.09	-216.30	-112.00
Maternal place of residence					
City	Referent				
Remote village		0.89	-99.35	-161.80	0.00
**At birth**					
Gestational age		0.99	-71.16	-104.70	-41.20
Placenta weight		0.85	-40.37	-69.00	0.00

STAGE 3 MODELS

Stage 3 model, outcome variable: mean BMI velocity between adiposity peak & adiposity rebound (BMIAPAR)

Model		Mean pp (γ)	Direct effect β (x10^{-3})	95% LCI	95% UCI
**Prenatal**					
Maternal BMI		0.77	69.60	0.00	114.30
Maternal marital status at birth					
Not married	Referent				

Married		1.00	106.34	-62.20	277.40
Maternal place of residence					
City	Referent				
Small town & rural centre		1.00	-106.81	-196.60	-9.50
Remote village		0.77	-127.35	-231.00	0.00
Number of people in the household		0.77	-58.77	-104.60	0.00
**At birth**					
Operative managements in delivery					
Vaginal	Referent				
Caesarean section		0.54	41.16	-11.90	164.90
**At infancy**					
Mean BMI growth velocity between birth & AP		0.77	-38.90	-75.70	0.00
BMI at AP		1.00	-281.44	-316.20	-248.20
Age at AP		1.00	176.85	147.10	207.70
Peak height velocity		0.77	74.08	0.00	124.70

Stage 3 model, outcome variable: mean BMI growth velocity between adiposity rebound & 11y (BMI AR-11)

Model		Mean pp (γ)	Direct effect β (x10^{-3})	95% LCI	95% UCI
**Prenatal**					
Maternal BMI		1.00	156.42	123.70	189.10
Parity		1.00	-89.07	-137.90	-37.30
Maternal marital status at birth					
Not married	Referent				
Married		0.54	-50.49	-241.10	48.40
Number of people in the household		0.23	-11.03	-70.70	0.00
**At birth**					
BMI PRS		1.00	59.83	34.00	85.60
Placenta weight		0.54	-7.79	-45.20	17.30
Birth weight		0.77	34.57	-0.10	81.30
**At infancy**					
BMI at AP		1.00	105.49	70.80	138.40
Age at AP		0.77	33.24	0.00	67.40
Peak height velocity		0.77	58.45	0.00	104.50

Stage 3 model, outcome variable: BMI at adiposity rebound (BMIAR)

Model		Mean pp (γ)	Direct effect β (x10^{-3})	95% LCI	95% UCI
**Prenatal**					
Number of people in the household		0.46	-44.65	-122.84	0.00
Maternal hypertension					
Normotensive	Referent				
Could not be determined/ Not known		0.54	17.77	-42.70	110.19

**At birth**					
BMI PRS		1.00	51.49	29.00	73.00
Gestational age		0.77	-1.13	-31.40	28.70
Placenta weight		0.54	-1.78	-30.10	23.00
Birth weight		0.77	69.67	0.00	119.70
**At infancy**					
BMI at AP		1.00	565.73	533.30	600.90
Age at AP		1.00	162.65	134.00	189.60
Peak height velocity		1.00	110.40	77.90	141.90

Stage 3 model, outcome variable: age at adiposity rebound (AgeAR)

Model		Mean pp (γ)	Direct effect β (x10^{-3})	95% LCI	95% UCI
**Prenatal**					
Maternal BMI		1.00	-175.44	-210.20	-141.40
Maternal smoking					
Non-smoker	Referent				
Continued/stopped		1.00	-96.21	-177.40	-16.80
Number of people in the household		1.00	108.46	70.50	145.50
Maternal hypertension					
Normotensive	Referent				
Gestational hypertension		1.00	-28.99	-142.50	81.70
**At birth**					
BMI PRS		0.77	-51.04	-87.20	0.00
**At infancy**					
BMI at AP		1.00	-68.80	-103.60	-34.20
Peak height velocity		0.77	-91.25	-148.30	0.00

STAGE 4 MODELS

Stage 4 model, outcome variable: Mean BMI velocity between 11y and 15 y (BMI 11-15)

Model		Mean pp (γ)	Direct effect β (x10^{-3})	95% LCI	95% UCI
**Prenatal**					
Maternal hypertension					
Normotensive	Referent				
Chronic hypertension		1.00	29.21	-123.60	180.80
Could not be determined/ Not known		0.53	15.02	-72.70	131.40
**At childhood (6 y)**					
Mean BMI growth velocity between AR & 11y		1.00	-586.32	-650.70	-519.70
Age at AR		1.00	-455.91	-518.60	-394.30

Stage 4 model, outcome variable: BMI at 14 years

Model		Mean pp (γ)	Direct effect β ($x10^{-3}$)	95% LCI	95% UCI
**Prenatal**					
Maternal BMI		0.54	9.25	-4.60	37.00
Maternal age (years)		0.24	-6.04	-37.00	0.00
**At birth**					
Placenta weight		0.98	5.19	-17.70	28.30
**At childhood (6 y)**					
Mean BMI growth velocity between AR & 11y		1.00	101.59	59.60	142.90
BMI at AR		1.00	346.84	316.30	376.50
Age at AR		1.00	-393.96	-435.50	-353.30
**At adolescence (14 y)**					
Smoking at 14y		0.53	-0.12	-52.70	50.90

STAGE 5 MODELS

Stage 5 model, outcome variable: Blood Pressure (latent) factor at 31y (BPF-31)

Model		Mean pp (γ)	Direct effect β ($x10^{-3}$)	95% LCI	95% UCI
**Prenatal**					
Maternal hypertension					
Normotensive	Referent				
Pre-eclampsia (PE) & Super-imposed PE		0.56	73.57	-49.70	314.60
**At adolescence (14 y)**					
BMI at 14y		0.78	97.96	0.00	158.40

Stage 5 model, outcome variable: BMI at 31y (BMI-31)

Model		Mean pp (γ)	Direct effect β ($x10^{-3}$)	95% LCI	95% UCI
**Prenatal**					
Maternal age (years)		0.56	-14.78	-51.50	0.10
Maternal place of residence					
City	Referent				
Remote village		0.56	32.91	-0.10	107.40
Maternal hypertension					
Normotensive	Referent				
Diastolic BP elevated		0.56	13.79	-63.90	113.70
**At infancy (1yr)**					
BMI at AP		0.56	67.68	0.00	144.20
**At childhood (6 y)**					
Mean BMI growth velocity between AP & AR		0.56	52.64	0.00	116.60
Mean BMI growth velocity between AR & 11y		1.00	175.75	72.00	290.40
Age at AR		1.00	-316.49	-393.90	-230.10
**At adolescence (14 y)**					

Physical activity at 14y	0.56	3.25	-53.10	65.70
**At early adulthood (31 y)**				
Smoking pack years at 31y	0.56	-4.46	-33.80	18.60
Alcohol use at 31y	0.78	25.71	-0.05	65.50

Stage 5 model, outcome variable: Insulin at 31y (INS-31)

Model		Mean pp (γ)	Direct effect β (x10^{-3})	95% LCI	95% UCI
**At birth**					
Birth weight		0.56	-33.03	-89.90	0.00
**At childhood (6 y)**					
BMI at AR		0.78	68.71	0.00	117.30

Stage 5 model, outcome variable: Waist Circumference at 31y (WC-31)

Model		Mean pp (γ)	Direct effect β (x10^{-3})	95% LCI	95% UCI
**Prenatal**					
Maternal hypertension					
Normotensive	Referent				
Chronic hypertension		0.78	-6.96	-135.30	121.30
**At birth**					
Operative managements in delivery					
Vaginal	Referent				
Caesarean section		0.78	-18.86	-131.58	81.10
Birth weight		0.64	23.67	-0.01	68.40
**At childhood (6 y)**					
Age at AR		0.56	-177.39	-340.30	0.00

Stage 5 model, outcome variable: HDL at 31y (HDL-31)

Model		Mean pp (γ)	Direct effect β (x10^{-3})	95% LCI	95% UCI
Smoking at 14y		1.00	-58.27	-160.50	42.20
BMI at 14y		0.56	-48.19	-110.80	0.00
**At early adulthood (31 y)**					
Smoking at 31y		0.78	-82.97	-163.10	0.00

Stage 5 model, outcome variable: LDL at 31y (LDL-31)

Model		Mean pp (γ)	Direct effect β (x10^{-3})	95% LCI	95% UCI
**Prenatal**					
SEP of the family		0.56	12.33	-10.10	52.40
Maternal hypertension					
Normotensive	Referent				
Pre-eclampsia (PE) & Super-imposed PE		1.00	65.82	-134.40	262.40
**At early adulthood (31y)**					

Smoking pack years at 31y	1.00	-8.40	-44.90	27.20

Stage 5 model, outcome variable: Triglycerides at 31y (TRIGL-31)

Model		Mean pp (γ)	Direct effect β (x10^{-3})	95% LCI	95% UCI
**Prenatal**					
Maternal hypertension					
Normotensive	Referent				
Chronic hypertension		0.56	13.60	-102.50	156.30
**At early adulthood (31y)**					
SEP latent factor-1 at 31y		0.56	27.23	-0.01	72.22

STAGE 6 MODEL

Stage 6 model, outcome variable: BMI at 46y (BMI46)

Model		Mean pp (γ)	Direct effect β (x10^{-3})	95% LCI	95% UCI
**Prenatal**					
Maternal BMI		0.48	12.45	-2.60	54.00
Maternal marital status at birth					
Not married	Referent				
Married		0.74	-31.64	-206.10	118.10
Maternal place of residence					
City	Referent				
Small town & rural centre		0.79	13.54	-44.40	77.10
Paternal age (years)		0.73	-19.04	-58.10	5.70
**At birth**					
Placenta weight		0.73	5.95	-21.80	37.80
**At adolescence (14 y)**					
Physical activity at 14y		1.00	22.59	-52.60	97.00
**At early adulthood (31 y)**					
BP latent factor at 31y		1.00	80.38	46.10	113.80
BMI at 31y		1.00	539.27	500.30	577.90
Insulin at 31y		0.85	25.49	-8.20	66.30
Triglycerides at 31y		0.59	10.37	-15.60	49.60
**At late adulthood (46 y)**					
SEP factor at 46y		1.00	-19.83	-48.40	7.00

Table S4: Posterior mean estimates (%) and 95% CI of the Bayesian version of the R2 showing how much of the variation in each of the endogenous variable is explained by dependence on the exogenous variables.

Bayesian R^2		
Endogenous variable	Posterior mean (%)	(95% CI)
Birth weight	34.5	(32.0,37.0)
Mean BMI growth velocity between birth & AP	50.7	(48.8,52.5)
BMI at AP	14.7	(12.8,16.7)
Age at AP	1.8	(1.0,2.6)
Peak height velocity	34.8	(32.6,36.9)
Mean BMI growth velocity between AP & AR	13.3	(11.0,15.3)
Mean BMI growth velocity between AR & 11y	6.4	(4.9,8.0)
BMI at AR	38.1	(36.0,40.3)
Age at AR	6.6	(4.9,8.2)
Mean BMI velocity 11-15 years	11.4	(9.3,13.5)
BMI at 14y	58.1	(56.3,59.8)
BP latent factor at 31y	7.5	(5.0,9.7)
BMI at 31y	32.3	(29.7,35.0)
Insulin at 31y	1.1	(0.0,2.1)
WC at 31y	24.0	(21.1,26.8)
HDL at 31y	10.8	(8.5,13.1)
LDL at 31y	4.1	(3.0,5.9)
Triglycerides	3.9	(2.4,5.9)
BMI at 46y	34.6	(31.5,37.6)

Abbreviations: CI: credible intervals; BMI: body mass index; AP: adiposity peak; AR: adiposity rebound; BP: blood pressure; WC: waist circumference; HDL: high density lipoprotein cholesterol; LDL: low density lipoprotein.

Model fitting procedure

Visual checks (density plots and Q-Q plots) were used to check normality assumptions for continuous variables. All endogenous variables had close to normal distributions except for the biomarkers at 31y which were log-transformed. One extreme observation for insulin (31y) was set to missing after checking for outliers. The relationship of birth weight with adult BMI was found to be reasonably linear.

Bayesian estimates of all parameters in the path analysis model were obtained after 150 000 Monte Carlo Markov Chain (MCMC) iteration runs for 3 multiple chains and after 20 000 iterations burn-in (BLSEM software). MCMC diagnostic trace plots were checked for model convergence. Given the complexity of these models, sensitivity analysis was conducted on subsamples of variables and life stages. Univariate analyses were performed for each endogenous variable. Bayesian BLSEM using smaller numbers of life stages were also run, and examined and compared with the full model to understand the interplay among the different variables. The models were fitted to the full cohort and to different groups of individuals based on the completeness of the stage specific outcomes (endogenous variables) (data not shown). Overall, our results highlighted the sensitivity of posterior estimates to missing values of the endogenous variables. The assumptions concerning the missing data mechanism remained the same throughout missing at random (MAR).

Figure S4: Subgraph of the Directed Acyclic Graph (DAG) from the Bayesian Path Analysis Model, BLSEM, showing all paths thresholding for mean posterior probabilities (MPPIs) ≥0.5 between maternal BMI (matBMI) and BMI46.

Direct effect*=0; total indirect effect*=52.3 (95% CI: 41.7,63.0); 35 pathways

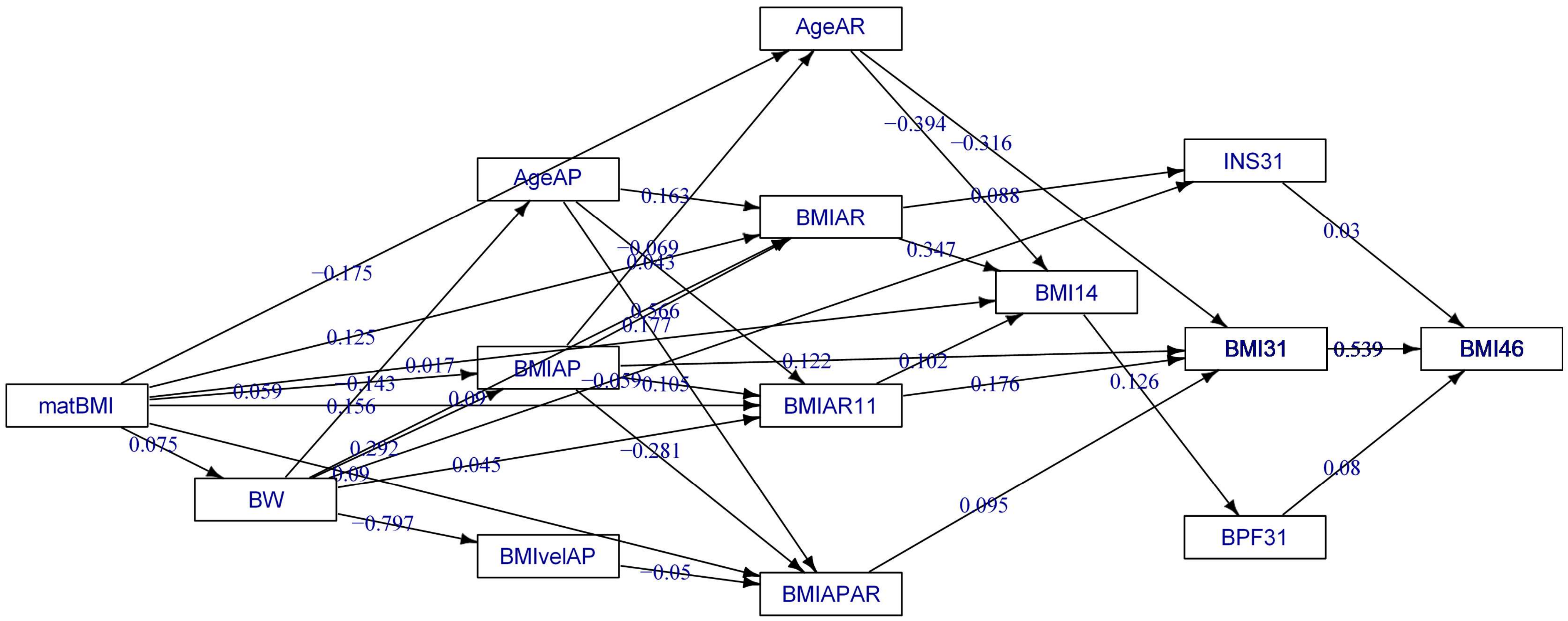

*The pairwise standardised effects (βs) x10 $^{-3}$ are shown on the edges interpreted as changes in SD units in the outcome per 1-SD change in the predictor or a change from 0 to 1 for binary predictors.

Figure S5: Subgraph of the Directed Acyclic Graph (DAG) from the Bayesian Path Analysis Model, BLSEM, showing all paths thresholding for mean posterior probabilities (MPPIs) ≥0.5 between maternal smoking (matSMO) and BMI46.

Direct effect*=0; total indirect effect*=22.5 (95% CI: 5.34, 41.0); 31 pathways

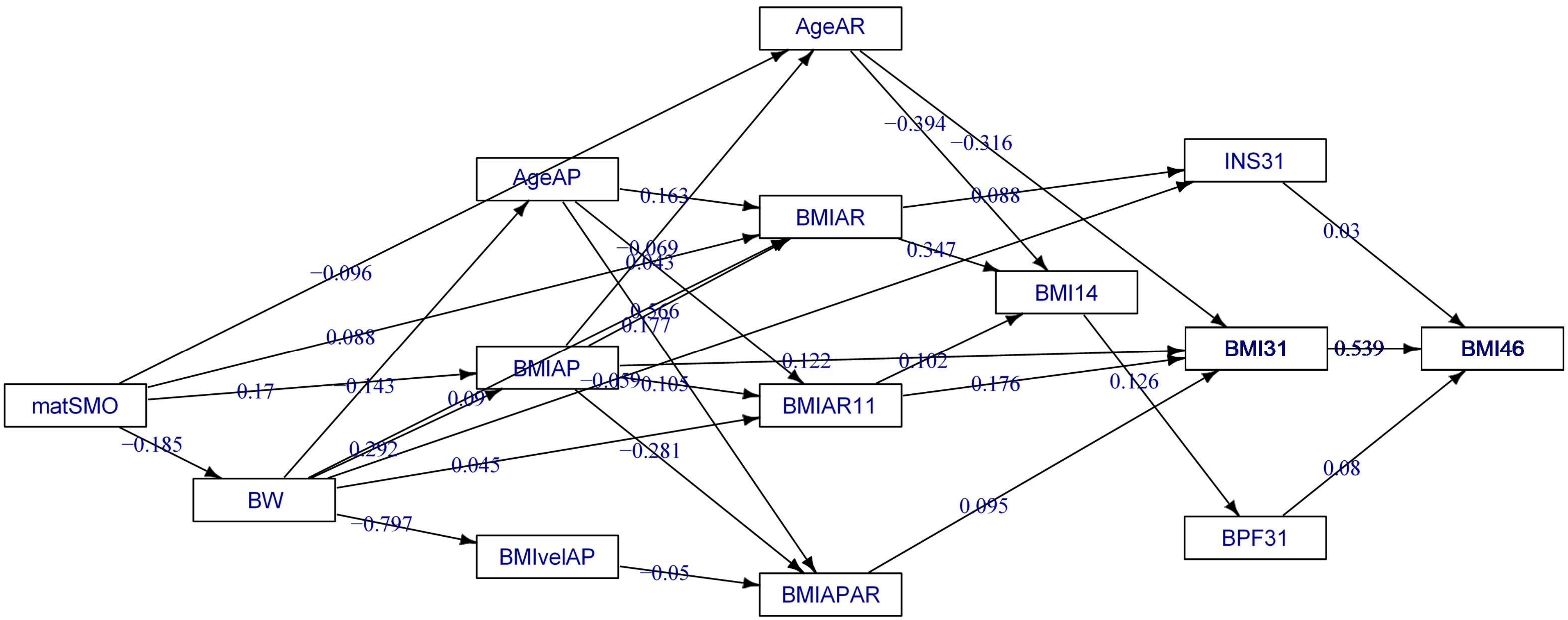

*The pairwise standardised effects (βs) x10 $^{-3}$ are shown on the edges interpreted as changes in SD units in the outcome per 1-SD change in the predictor or a change from 0 to 1 for binary predictors.

Figure S6: Subgraph of the Directed Acyclic Graph (DAG) from the Bayesian Path Analysis Model, BLSEM, showing all paths thresholding for mean posterior probabilities (MPPIs) ≥0.5 between maternal SEP (matSEP) and BMI46. Better maternal socioeconomic position associated with lower middle-age BMI.

Direct effect*=0; total indirect effect*=-1.85 (95% CI: -3.57,-0.18); 26 pathways

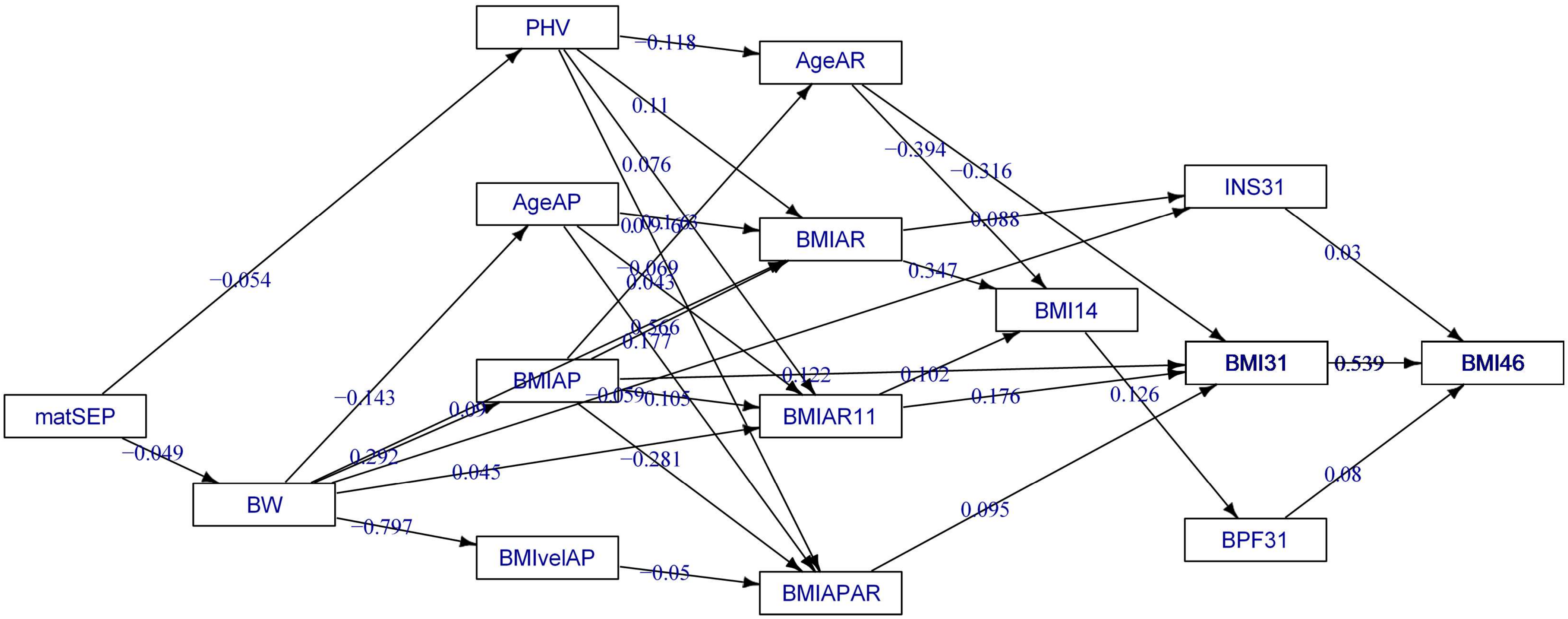

*The pairwise standardised effects (βs) x10 $^{-3}$ are shown on the edges interpreted as changes in SD units in the outcome per 1-SD change in the predictor or a change from 0 to 1 for binary predictors.

Figure S7: Subgraph of the Directed Acyclic Graph (DAG) from the Bayesian Path Analysis Model, BLSEM, showing all paths thresholding for mean posterior probabilities (MPPIs) ≥0.5 between birth weight (BW) and BMI-46.

Direct effect*=0; total indirect effect*=17.9(95% CI: 5.5, 29.3); 19 pathways

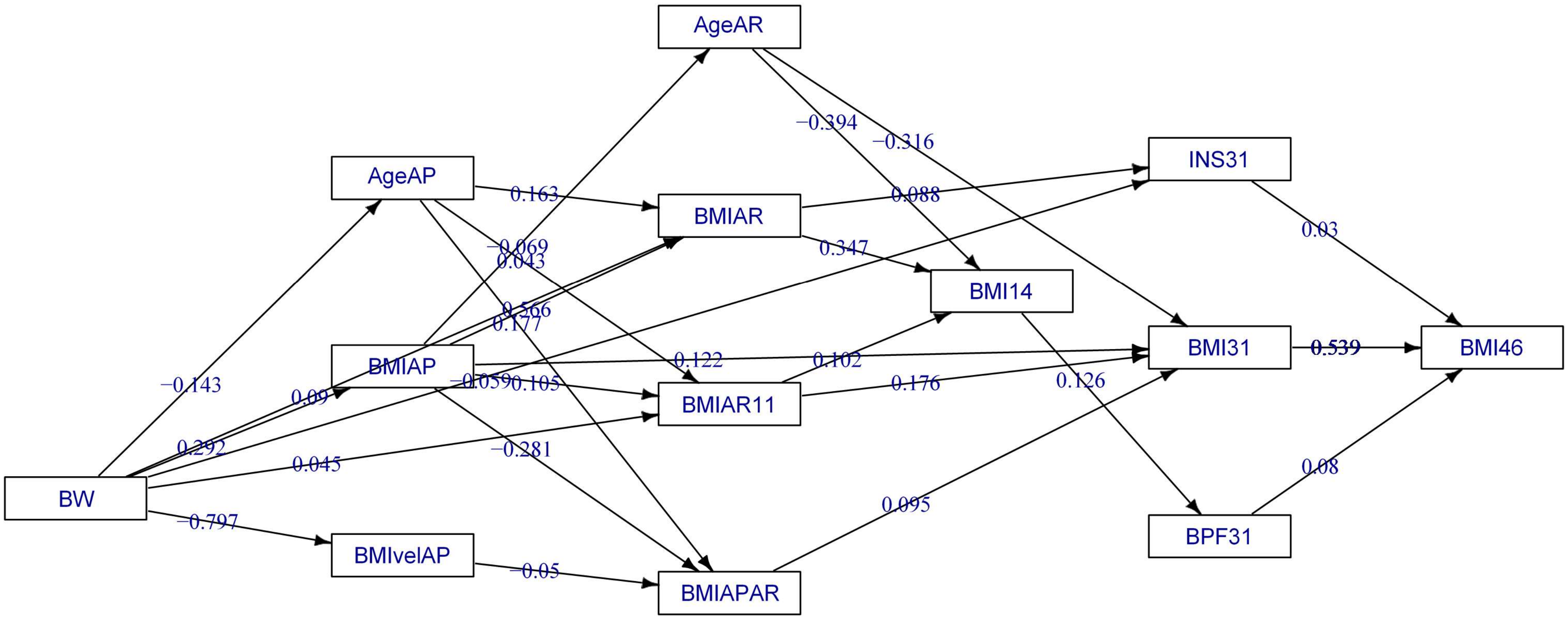

*The pairwise standardised effects (βs) x10^{-3} are shown on the edges interpreted as changes in SD units in the outcome per 1-SD change in the predictor or a change from 0 to 1 for binary predictors.

Figure S8: Subgraph of the Directed Acyclic Graph (DAG) from the Bayesian Path Analysis Model, BLSEM, showing all paths thresholding for mean posterior probabilities (MPPIs) ≥0.5 between age at AR (AgeAR) and BMI46.

Direct effect*=0; total indirect effect*=-174.0 (95% CI: -219.0,-67.8); 2 pathways

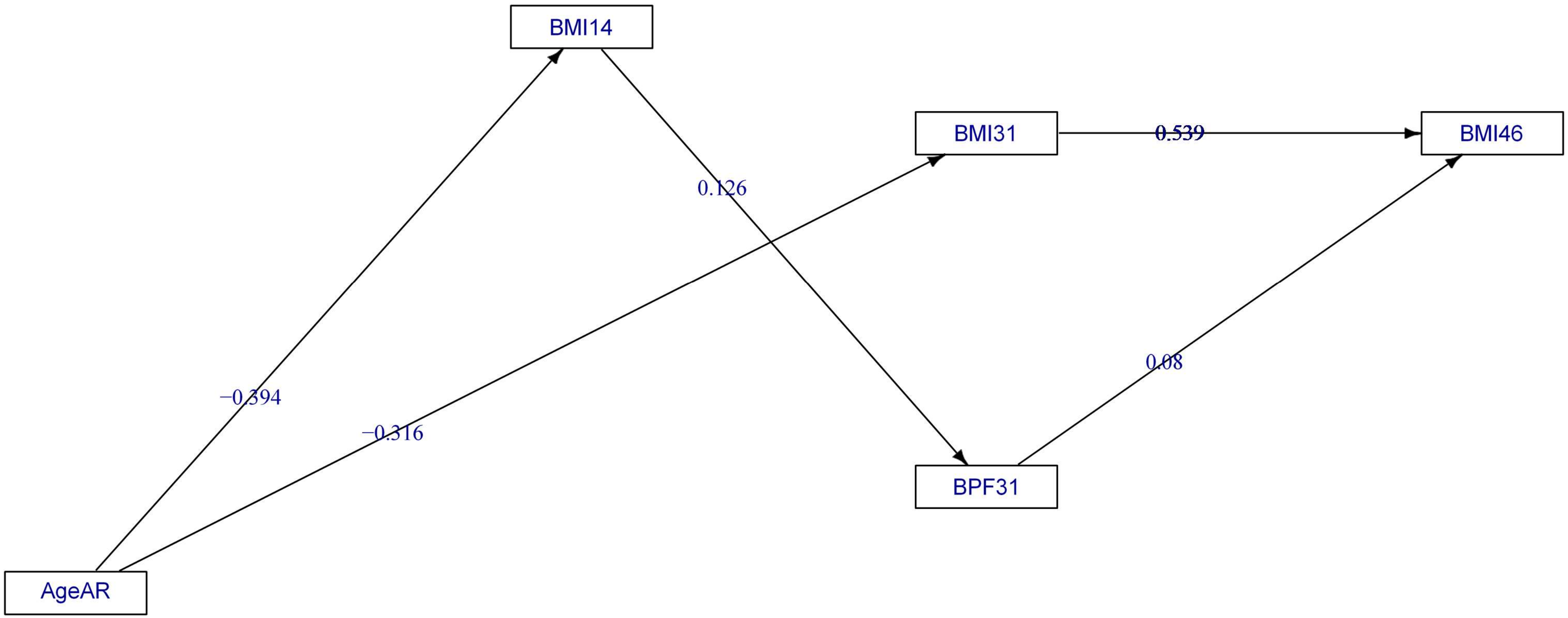

*The pairwise standardised effects (βs) x10 $^{-3}$ are shown on the edges interpreted as changes in SD units in the outcome per 1-SD change in the predictor or a change from 0 to 1 for binary predictors.

Figure S9: Subgraph of the Directed Acyclic Graph (DAG) from the Bayesian Path Analysis Model, BLSEM, showing all paths thresholding for mean posterior probabilities (MPPIs) ≥0.5 between Mean BMI velocity AR-11y (BMIAR11) and BMI46.

Direct effect*=0; total indirect effect*=95.6 (95% CI: 39.1,158.0); 2 pathways

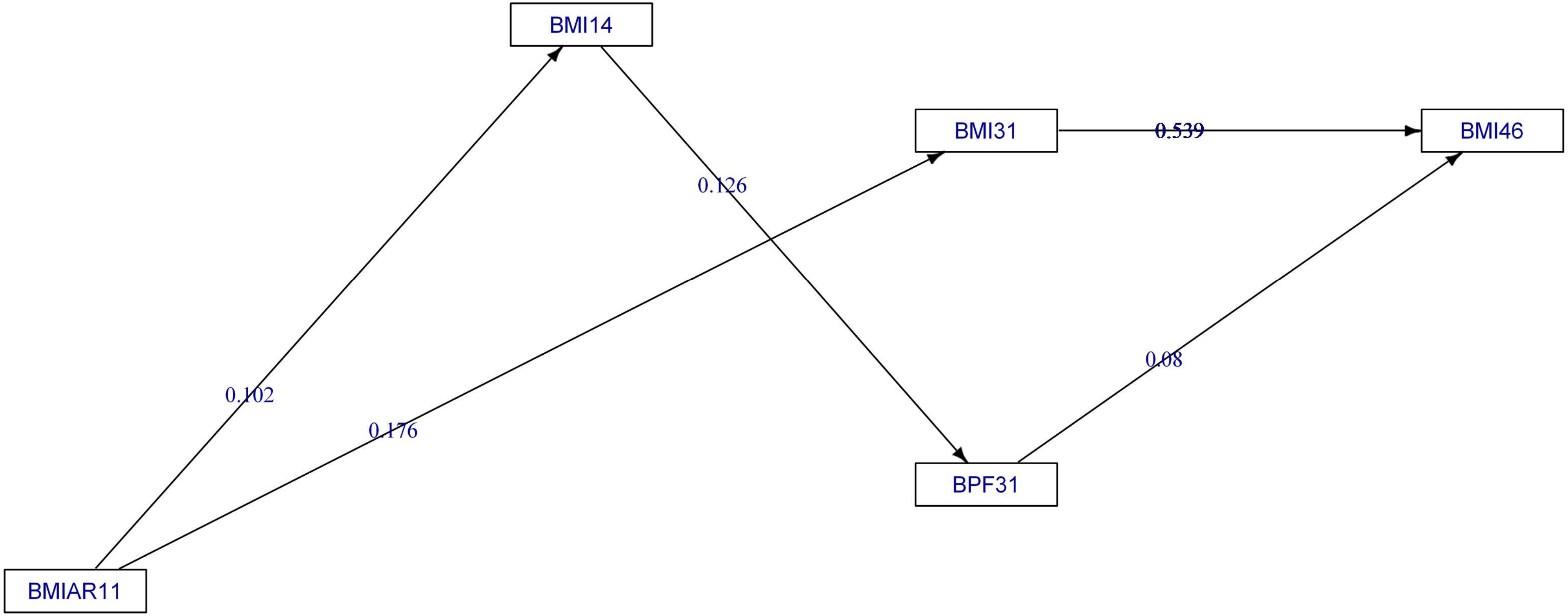

*The pairwise standardised effects (βs) x10 $^{-3}$ are shown on the edges interpreted as changes in SD units in the outcome per 1-SD change in the predictor or a change from 0 to 1 for binary predictors.

Figure S10: Subgraph of the Directed Acyclic Graph (DAG) from the Bayesian Path Analysis Model, BLSEM, showing all paths thresholding for mean posterior probabilities (MPPIs) ≥0.5 between BMI at AP (BMIAP) and BMI46.

Direct effect*=0; total indirect effect*=53.1 (95% CI: 18.7,89.4); 8 pathways;

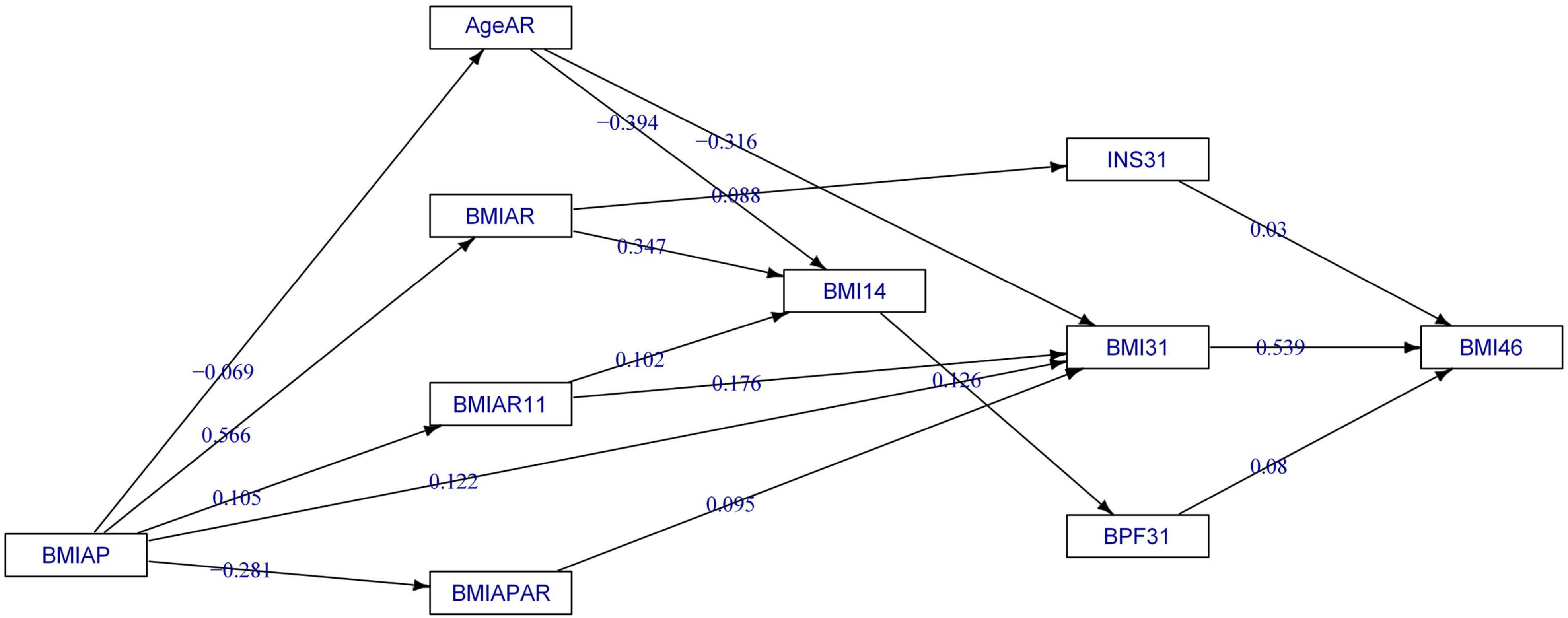

*The pairwise standardised effects (βs) x10 $^{-3}$ are shown on the edges interpreted as changes in SD units in the outcome per 1-SD change in the predictor or a change from 0 to 1 for binary predictors.

References

1. Nordström T, Miettunen J, Auvinen J, Ala-Mursula L, Keinänen-Kiukaanniemi S, Veijola J, et al. Cohort Profile: 46 years of follow-up of the Northern Finland Birth Cohort 1966 (NFBC1966). Int J Epidemiol. 2022 Jan 6;50(6):1786–1787j.

2. Näyhä S, Lankila T, Rautio A, Koiranen M, Tammelin TH, Taanila A, et al. Body mass index and overweight in relation to residence distance and population density: experience from the Northern Finland birth cohort 1966. BMC Public Health. 2013 Dec 8;13(1):938.

3. Tzoulaki I, Sovio U, Pillas D, Hartikainen AL, Pouta A, Laitinen J, et al. Relation of Immediate Postnatal Growth With Obesity and Related Metabolic Risk Factors in Adulthood: The Northern Finland Birth Cohort 1966 Study. Am J Epidemiol. 2010 May 1;171(9):989–98.

4. Sovio U, Kaakinen M, Tzoulaki I, Das S, Ruokonen A, Pouta A, et al. How do changes in body mass index in infancy and childhood associate with cardiometabolic profile in adulthood? Findings from the Northern Finland Birth Cohort 1966 Study. Int J Obes. 2014 Jan 4;38(1):53–9.

5. Nedelec R, Miettunen J, Männikkö M, Järvelin MR, Sebert S. Maternal and infant prediction of the child BMI trajectories; studies across two generations of Northern Finland birth cohorts. Int J Obes. 2021 Feb 11;45(2):404–14.

6. Rantakallio P. Family Background to and Personal Characteristics Underlying Teenage Smoking. Scand J Soc Med. 1983 Mar 27;11(1):17–22.

7. Tammelin T, Laitinen J, Näyhä S. Change in the level of physical activity from adolescence into adulthood and obesity at the age of 31 years. Int J Obes. 2004 Jun 23;28(6):775–82.

8. Lowry E, Rautio N, Karhunen V, Miettunen J, Ala-Mursula L, Auvinen J, et al. Understanding the complexity of glycaemic health: systematic bio-psychosocial modelling of fasting glucose in middle-age adults; a DynaHEALTH study. Int J Obes. 2019 Jun 17;43(6):1181–92.

9. Isohanni I, Järvelin MR, Rantakallio P, Jokelainen J, Jones PB, Nieminen P, et al. Juvenile and early adulthood smoking and adult educational achievements — A 31-year follow-up of the Northern Finland 1966 Birth Cohort. Scand J Public Health. 2001 Apr 5;29(2):87–95.

10. Laitinen J, Pietiläinen K, Wadsworth M, Sovio U, Järvelin MR. Predictors of abdominal obesity among 31-y-old men and women born in Northern Finland in 1966. Eur J Clin Nutr. 2004 Jan 1;58(1):180–90.

11. Jääskeläinen A, Kaila-Kangas L, Leino-Arjas P, Lindbohm ML, Nevanperä N, Remes J, et al. Association between occupational psychosocial factors and waist circumference is modified by diet among men. Eur J Clin Nutr. 2015 Sep 22;69(9):1053–9.

12. Järvelin MR, Sovio U, King V, Lauren L, Xu B, McCarthy MI, et al. Early Life Factors and Blood Pressure at Age 31 Years in the 1966 Northern Finland Birth Cohort. Hypertension. 2004 Dec;44(6):838–46.

13. Sabatti C, Service SK, Hartikainen AL, Pouta A, Ripatti S, Brodsky J, et al. Genome-wide association analysis of metabolic traits in a birth cohort from a founder population. Nat Genet. 2009 Jan 7;41(1):35–46.

14. McCarthy Shane, Das Sayantan, Kretzschmar Warren, DelaneauOlivier, Wood Andrew R, Teumer Alexander, et al. A reference panel of 64,976 haplotypes for genotype imputation. Nat Genet. 2016 Oct 22;48(10):1279–83.

15. Das S, Forer L, Schönherr S, Sidore C, Locke AE, Kwong A, et al. Next-generation genotype imputation service and methods. Nat Genet. 2016 Oct 29;48(10):1284–7.

16. Gelman A, Goodrich B, Gabry J, Vehtari A. R-squared for Bayesian Regression Models. Vol. 73, American Statistician. 2019.

17. Bottolo L, Chadeau-Hyam M, Hastie DI, Langley SR, Petretto E, Tiret L, et al. *ESS* ++: a C++ objected-oriented algorithm for Bayesian stochastic search model exploration. Bioinformatics. 2011 Feb 15;27(4):587–8.

18. VanderWeele T, Vansteelandt S. Mediation Analysis with Multiple Mediators. Epidemiol Methods. 2014 Jan 3;2(1).

19. Guan Y, Stephens M. Bayesian variable selection regression for genome-wide association studies and other large-scale problems. Ann Appl Stat. 2011 Sep 1;5(3).

20. Wang Y, Chen Z, Goldstein JM, Buck Louis GM, Gilman SE. A Bayesian regularized mediation analysis with multiple exposures. Stat Med. 2019 Feb 28;38(5):828–43.

21. Song Y, Zhou X, Zhang M, Zhao W, Liu Y, Kardia SLR, et al. Bayesian shrinkage estimation of high dimensional causal mediation effects in omics studies. Biometrics. 2020 Sep 19;76(3):700–10.

22. Huang Y, Pan W. Hypothesis test of mediation effect in causal mediation model with high-dimensional continuous mediators. Biometrics. 2016 Jun 28;72(2):402–13.
